# The Contribution of Premotor Cortico-Striatal Projections to the Execution of Serial Order Sequences

**DOI:** 10.1523/ENEURO.0173-21.2021

**Published:** 2021-09-21

**Authors:** Asai Sánchez-Fuentes, Kathia I. Ramírez-Armenta, Anil Kumar Verma-Rodríguez, Edgar Díaz-Hernández, Antonio Aguilar-Palomares, Josué O. Ramírez-Jarquín, Fatuel Tecuapetla

**Affiliations:** Instituto de Fisiología Celular, Universidad Nacional Autónoma de México, Ciudad de México 04510, México

**Keywords:** action sequences, cortico-striatal, motor cortex, optogenetics, premotor cortex, synapses

## Abstract

Striatal activity is necessary to initiate and execute sequences of actions. The main excitatory input to the striatum comes from the cortex. While it is hypothesized that motor and premotor cortico-striatal projections are important to guide striatal activity during the execution of sequences of actions, technical limitations have made this challenging to address. Here, we implemented a task in mice that allows for the study of different moments to execute a serial order sequence consisting of two subsequences of actions. Using this task, we performed electrophysiological recordings in the premotor (M2) and primary motor (M1) cortices, and state-dependent optogenetic inhibitions of their cortico-striatal projections. We show that while both M2 and M1 contain activity modulations related to the execution of self-paced sequences, mainly, the premotor cortico-striatal projections contribute to the proper execution/structuring of these sequences.

## Significance Statement

It is currently hypothesized that synapses from the primary motor (M1) and premotor (M2) cortices that innervate the striatum may guide the proper execution of sequences. Here, we evaluated this hypothesis by training animals to execute self-paced sequences: performing recordings in M2-M1 or manipulating their cortico-striatal projections during the execution of these sequences. We show that both, M2-M1 cortico-striatal projections contribute to sequence initiation, however sequence execution is predominantly influenced by M2. Remarkably the contribution of the cortico-striatal projections from M2 is mainly before the initiation of the sequence working to sustain the structure of the sequences, mainly during the beginning. These findings may have implications for pathologic conditions where the self-paced generation of sequences of actions is impaired.

## Introduction

In everyday life, we continuously move between sequences of motor actions. One of the main proposed drivers involved in the learning and execution of motor sequences are cortico-striatal projections. The study of action sequences in relation to cortico-striatal function has become increasingly important since the discovery that symptoms in patients with Parkinson’s disease and obsessive-compulsive disorder maybe be caused by disruptions to cortico-striatal projections (Graybiel, 1998; Redgrave et al., 2010; Burguière et al., 2013).

The striatum, the primary input to basal ganglia (BG), is a subcortical structure whose activity is necessary to initiate and execute a sequence of actions (Cui et al., 2013; Jin et al., 2014; Rothwell et al., 2015; Tecuapetla et al., 2016; Dhawale et al., 2021). Recent evidence suggests that a specific subcircuit within the BG, the indirect pathway, is essential for the transition between subsequences (Geddes et al., 2018; Tecuapetla et al., 2016).

The striatum’s main glutamatergic inputs come from the cortex and the thalamus (Wilson, 1989). Several studies suggest that the cortical inputs are essential to execute motor sequences (Tanji and Evarts, 1976; Inase et al., 1996; Martiros et al., 2018; Shima and Tanji, 2000; Fujii and Graybiel, 2005; Shima et al., 2007; Burguière et al., 2013; Smith and Graybiel, 2013; Friedman et al., 2015; Rothwell et al., 2015; Kupferschmidt et al., 2017; Martiros et al., 2018). However, the specific contribution of cortico-striatal projections to the execution of self-paced action sequences remains unclear.

To date, it is known that the supplementary motor area in primates (SMA), which corresponds to the secondary motor cortex (M2) in rodents, is active before starting a sequence of actions (Donoghue and Wise, 1982; Romo and Shultz 1987; Passingham et al., 1988; Shima et al., 2007; Nakayama et al., 2008). SMA activity is important to adapt the behavior in response to contingency changes (block changes; Hikosaka and Isoda, 2010; Siniscalchi et al., 2016). These findings have led to the hypothesis that the premotor cortex guides the striatal activity to initiate and execute action sequences. In rodents, a decrease in the activity of neurons in M2 decreases the probability of alternating between two actions (Rothwell et al., 2015). Strikingly however, studies using cortical lesions suggest that the striatum (BG) can control the execution of a sequence of actions independently from the cortex (Kawai et al., 2015; Dhawale et al., 2021). Therefore, we implemented a self-paced serial order sequence task that allows for probing the contribution of M2 cortico-striatal projections during the initiation, execution, and transition between subsequences of actions. By recording neuronal activity in premotor (M2) and primary motor (M1) cortices and performing time-dependent optogenetic inhibitions of the cortico-striatal projections, we identified specific contributions of the premotor cortico-striatal projections to the execution of self-paced serial order action sequences. Our results support a model in which the cortico-striatal terminals from M2 guide the appropriate execution of self-paced sequences of actions.

## Materials and Methods

### Animals

The institutional committee of the Cell Physiology Institute, at the National Autonomous University of Mexico, approved all procedures for the care and use of laboratory animals (protocol number FT121-17). This protocol follows the National Norm for Animals’ use (NOM-062-ZOO-1999). Male and female mice from two to three months of age at the start of experiments were used for this study. Two genotypes were used: C57BL/6J (The Jackson Laboratory, RRID: IMSR_JAX_000664) or Emx1-Cre mice (targeting the Cre recombinase expression in pyramidal cortical neurons), which had been backcrossed into C57BL/6J for at least six generations (Gorski et al., 2002). Emx1-Cre parental line was donated by Professor Rui M. Costa from the Champalimaud Center for the Unknown (RRID: MGI_5141283). All animals were obtained from our breeding colony in our institutional bioterium (the Emx1-Cre line is maintained in heterozygosis). Animals are housed under a 12/12 h light/dark cycle (lights on at 6 A.M.) with *ad libitum* access to food and water before beginning behavioral experiments.

### Training

**Figure 1.**
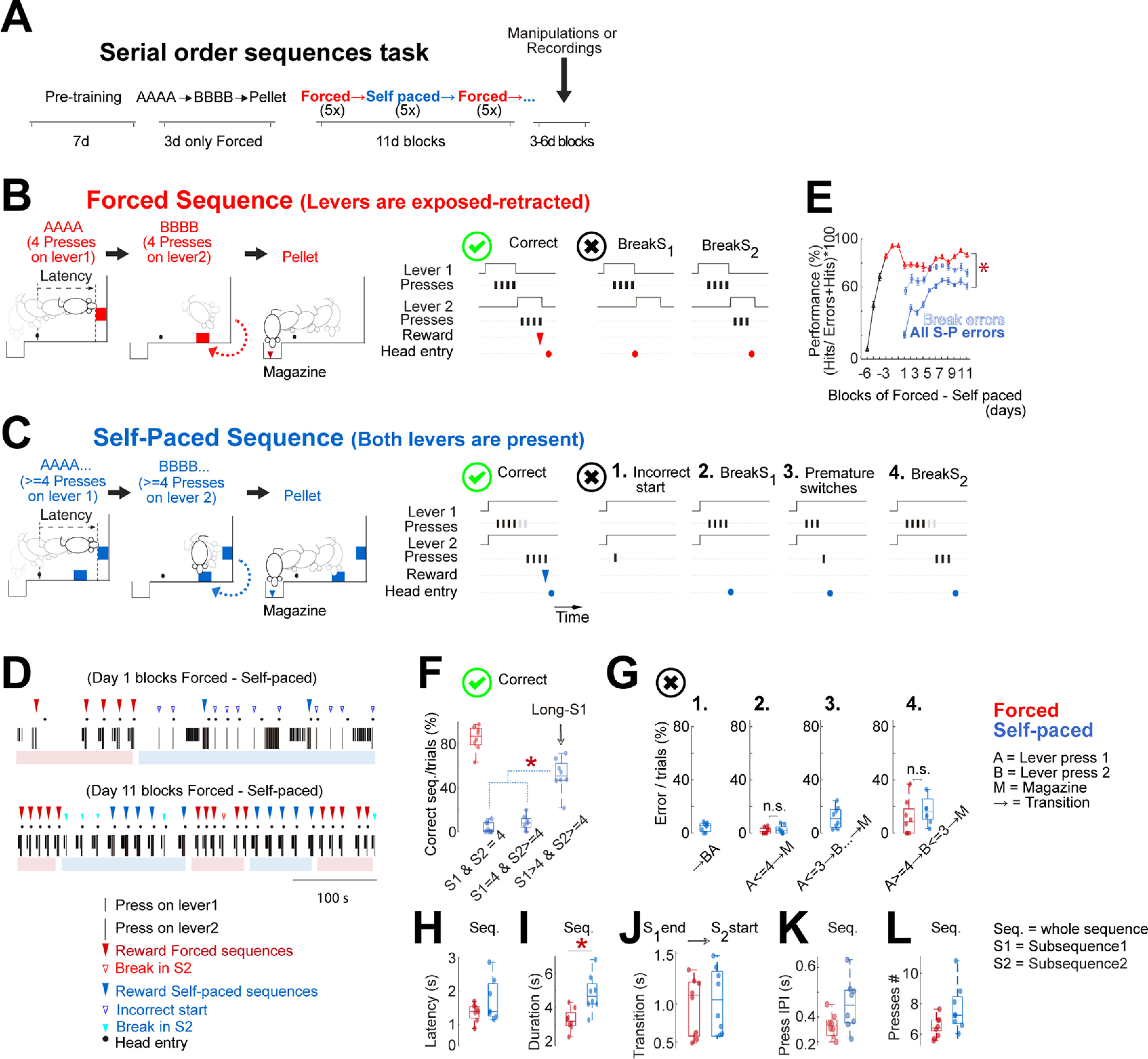
Mice learn to execute serial order sequences of lever press. ***A***, Timeline of training days. After 7 d of pretraining (see Materials and Methods), animals enter a phase of forced sequences (AAAA→BBBB→pellet). After 3 d, animals started training on blocks of forced and self-paced sequences, switching blocks once five correct trials are achieved per block. ***B***, left, Diagrams of the sequential pressing on lever 1 (AAAA = 4 presses) → lever 2 (BBBB = 4 presses) delivering a reward in the magazine (pellet) in forced sequences, where levers are presented individually. Right panels, Correct forced sequence depicted with a green symbol and the two different categories of errors that mice executed in forced sequences. ***C***, left, Diagrams of the sequential pressing on lever 1 (AAAA… >= 4 presses) → lever 2 (BBBB… >= 4 presses) delivering a reward in the magazine (pellet) in self-paced sequences, where levers are presented together. Right panels, Example of a correct sequence depicted with a green symbol and the four categories of errors that mice executed while performing self-paced sequences. ***D***, Example of the sequences of one animal’s presses during the first day (early in training) and eleventh day (late in training) during sessions of blocks of forced–self-paced sequences. Note how the self-paced sequences become very stereotyped late in training [the blocks are indicated by light red (forced) or light blue (self-paced) at the bottom of each panel]. ***E***, Percentage of correct sequences [correct/(errors + correct)]. Note that day 1 in the *x*-axis means the first day in which animals were required to perform blocks of forced and self-paced sequences as explained in ***A–C***. However, the requirement of forced sequences (AAAA→BBBB→pellets) was present 3 d before. All curves show mean ± SEM WT (*n* = 8). ***F***, The correct forced sequences were of one type (S1 = 4→S2 = 4) but of several types for self-paced sequences, dominated by Long-S1 sequences (S > 4→S2 >= 4). ***G***, The different categories of errors that mice executed while performing forced or self-paced sequences late in training. ***H***, Latency to start, measured as the time between breaking the infrared beam placed outside the magazine and the first press in the sequence. ***I*–*L***, Mean duration, transition, interpress intervals, number of presses from WT animals (*n* = 8) measured late in training; *p < 0.05, specific *p* values and statistical test specified in the text and Table 1. Extended Data Figure 1-1 includes the latency, transition time, IPI along with training, and a comparison of the proportion of the different errors early and late in training. n.s. = *p* > 0.05.

We used operant conditioning boxes equipped with two retractable levers to implement forced and self-paced serial order sequences in mice (21.6 cm long × 17.8 cm wide × 12.7 cm high; Med-Associates, catalog #MED-307W-D1). One lever was positioned on the left panel (subsequence 1; S1), and the other was located on the front panel (subsequence 2; S2) on the left side of the magazine (Fig. 1*B*,*C*). A small sugar pellet, 14 mg (Bio-Serv, catalog #F05684), was delivered as a reward in the magazine. Entries to the magazine were registered with an infrared beam. A second infrared beam was positioned between the magazine and left lever press (Fig. 1*B*,*C*) to calculate the latency to start the sequences of actions. Mice were subjected to food restriction throughout training and given enough food after daily training sessions to keep them at 80–85% of their original weight, depending on performance.

10.1523/ENEURO.0173-21.2021.f1-1 Extended Data Figure 1-1 Extended data from Figure 1. ***A***, Latency to start the sequences (1), transition time between the last press in the subsequence 1 to the first press in the subsequence 2 (2), and the press intervals intrasequences (3) along days. ***B***, The different categories of errors that mice executed while performing forced or self-paced sequences early and late in training. ***C***, ***D***, Mean of duration and lever presses per individual subsequences. Data from WT animals (*n* = 8); *p < 0.05, Mann–Whitney *U* test. Download Figure 1-1, TIF file.

**Table 1:** Descriptive statistics and data extended from the main figures

	Panel/data	Descriptive statistics	Type of test	*p* value	*n*
Data related to Figure 4	Breaks S1	Mean ± SEM (%) Forced off vs forced on M2-LS: 0 ± 0 vs 1.8 ± 0.9; M1-LS: 0.8 ± 0.8 vs 2.3 ± 1.2; controls: 0 ± 0 vs 2.6 ± 1.4 Forced: change rate (on–off) M2-LS: 1.8 ± 0.9 vs controls: 2.6 ± 1.4 M1-LS: 1.4 ± 0.8 vs controls: 2.6 ± 1.4	Paired two-sided permutation *t* test Unpaired two-sided permutation *t* test	1; 1; 1 0.68 0.49	Emx1-cre mice (+/−) AAV-Arch-M2-LS: *n* = 9 AAV-Arch-M1-LS: *n* = 8 Control: AAV-eYFP-M2-LS: *n* = 9
	Breaks S2	Mean ± SEM (%) Forced off vs forced on M2-LS: 13.7 ± 3.3 vs 7.8 ± 2.9; M1-LS: 12 ± 6 vs 19 ± 7; controls: 17 ± 4 vs 14 ± 3 Forced: change rate (on–off) M2-LS: 5.8 ± 3 vs controls: −3 ± 2.6 M1-LS: 6.9 ± 6 vs controls: −3 ± 6.9	Paired two-sided permutation *t* test Unpaired two-sided permutation *t* test	0.1; 0.3; 0.2 0.84 0.79	
	Sequence	Mean ± SEM (# presses) Forced off vs forced on M2-LS: 8.3 ± 0.1 vs 8.4 ± 0.2; M1-LS: 8.1 ± 0.2 vs 8 ± 0.1; controls: 8.1 ± 0.1 vs 8 ± 0.1 Forced: change rate (on–off) M2-LS: 0.07 ± 0.3 vs controls: −0.03 ± 0.09 M1-LS: −0.08 ± 0.1 vs controls: −0.03 ± 0.09	Paired two-sided permutation *t* test Unpaired two-sided permutation *t* test	0.8; 0.6;0.7 0.84 0.79	
	Latency	Mean ± SEM (s) Forced off vs forced on M2-LS: 1.4 ± 0.1 vs 2.4 ± 0.3; M1-LS: 2.9 ± 0.6 vs 3.6 ± 0.6; controls: 2.0 ± 0.4 vs 1.8 ± 0.3 Forced: change rate (on–off) M2-LS: 1.0 ± 0.2 vs controls: −0.2 ± 0.2 M1-LS: 0.7 ± 0.6 vs controls: −0.2 ± 0.2	Paired two-sided permutation *t* test Unpaired two-sided permutation *t* test	0.0001***;0.23;0.3 0.0016** 0.11	
	Return to start	Mean ± SEM (%) Forced off vs forced on M2-LS: 0.7 ± 0.7 vs 6 ± 3; M1-LS: 2.7 ± 1 vs 13 ± 5; controls: 1 ± 1 vs 1 ± 0.7 Forced: change rate (on–off) M2-LS: 5.7 ± 2.9 vs controls: −0.06 ± 1 M1-LS: 10.7 ± 5.4 vs controls: −0.06 ± 1.9	Paired two-sided permutation *t* test Unpaired two-sided permutation *t* test	0.1;0.06;0.8 0.11 0.04*	
Data related to Extended Data Figure 4-1	Performance	Data presented in the figure Forced vs self-paced Forced vs S-P-break errors	Mann–Whitney *U* test	0.0001*** 0.32	C57BL6/J mice (WT) AAV-Synapsine-Arch-M2-LS: *n* = 8 Control group *n* = 16
	Latency Inhibition before	Mean ± SEM (s) Forced off vs forced on LS: 2.5 ± 0.2 vs 4.2 ± 0.6; controls: 2.5 ± 0.3 vs 2.1 ± 0.33 Forced: change rate (on–off) LS: 1.6 ± 0.8 vs controls: −0.3 ± 0.2	Paired two-sided permutation *t* test Mann–Whitney *U* test	0.02*; 0.3 0.003**	
	Breaks S1 Inhibition before	Mean ± SEM (%) Forced off vs forced on LS: 7.4 ± 4 vs 9 ± 4; controls: 0.8 ± 0.8 vs 1.8 ± 0.8 Forced: change rate (on–off) LS: 2 ± 2 vs controls: 0.9 ± 1.3	Paired two-sided permutation *t* test Unpaired two-sided permutation *t* test	0.38; 0.55 0.67	
	Breaks S2 Inhibition before	Mean ± SEM (%) Forced off vs forced on LS: 20 ± 6.2 vs 11 ± 3.7; controls: 17.7 ± 3.4 vs 14.9 ± 2.4 Forced: change rate (on–off) LS: −8.7 ± 3 vs controls: −2 ± 2.5	Paired two-sided permutation *t* test Unpaired two-sided permutation *t* test	0.06; 0.29 0.20	
	Long-S1 sequences Inhibition before	Mean ± SEM (%) Forced off vs forced on LS: 0 ± 0 vs 1.1 ± 1; controls: 1.8 ± 1 vs 0.9 ± 0.5 Forced: change rate (on–off) LS: 1.1 ± 1 vs controls: −0.8 ± 1.1	Paired two-sided permutation *t* test Unpaired two-sided permutation *t* test	1.0; 0.51 0.36	
	Transition Inhibition before	Mean ± SEM (s) Forced off vs forced on LS: 1.1 ± 0.1 vs 1.0 ± 0.09; controls: 1.0 ± 0.1 vs 1.0 ± 0.1 Forced: change rate (on–off) LS: −0.06 ± 0.1 vs controls: 0 ± 0.03	Paired two-sided permutation *t* test Unpaired two-sided permutation *t* test	0.69; 0.78 0.54	
	Presses Inhibition before	Mean ± SEM (# presses) Forced off vs forced on LS: 7.6 ± 0.09 vs 7.7 ± 0.08; controls: 8.0 ± 0.1 vs 7.9 ± 0.09 Forced: change rate (on–off) LS: 0.1 ± 0.06 vs controls: −0.08 ± 0.1	Paired two-sided permutation *t* test Unpaired two-sided permutation *t* test	0.05; 0.46 0.127	
	Latency Inhibition execution	Mean ± SEM (s) Forced off vs forced on LS: 1.6 ± 0.1 vs 1.4 ± 0.1; controls: 2.6 ± 0.2 vs 3.1 ± 0.2 Forced: change rate (on–off) LS: −0.2 ± 0.1 vs controls: 0.5 ± 0.1	Paired two-sided permutation *t* test Unpaired two-sided permutation *t* test	0.01*; 0.04* 0.001**	
	Breaks S1 Inhibition execution	Mean ± SEM (%) Forced off vs forced on LS: 2.9 ± 1.4 vs 6.6 ± 2.8; controls: 1.4 ± 0.6 vs 1.8 ± 0.9 Forced: change rate (on–off) LS: 3.6 ± 3 vs controls: 0.4 ± 0.9	Paired two-sided permutation *t* test Unpaired two-sided permutation *t* test	0.38; 0.74 0.30	
	Breaks S2 Inhibition execution	Mean ± SEM (%) Forced off vs forced on LS: 17.7 ± 4 vs 22.5 ± 5.8; controls: 19.9 ± 2.7 vs 18.8 ± 3.5 Forced: change rate (on–off) LS: 4.8 ± 6 vs controls: −1.1 ± 4.2	Paired two-sided permutation *t* test Mann–Whitney *U* test	0.48; 0.78 0.44	
	Long-S1 sequences Inhibition execution	Mean ± SEM (%) Forced off vs forced on LS: 0.7 ± 0.7 vs 0.8 ± 0.8; controls: 0.2 ± 0.2 vs 1.0 ± 0.8 Forced: change rate (on–off) LS: 0.1 ± 1.2 vs controls: 0.8 ± 0.5	Paired two-sided permutation *t* test Unpaired two-sided permutation *t* test	0.50; 1.0 0.52	
	Transition Inhibition execution	Mean ± SEM (s) Forced off vs forced on LS: 1.0 ± 0.1 vs 1.0 ± 0.07; controls: 1.1 ± 0.12 vs 1.1 ± 0.1 Forced: change rate (on–off) LS: 0.01 ± 0.07 vs controls: 0.04 ± 0.04	Paired two-sided permutation *t* test Unpaired two-sided permutation *t* test	0.83; 0.39 0.72	
	Presses Inhibition execution	Mean ± SEM (# presses) Forced off vs forced on LS: 7.6 ± 0.08 vs 7.6 ± 0.1; controls: 7.6 ± 0.04 vs 7.7 ± 0.06 Forced: change rate (on–off) LS: −0.09 ± 0.1 vs controls: 0.02 ± 0.07	Paired two-sided permutation *t* test Unpaired two-sided permutation *t* test	0.54; 0.78 0.47	
	Latency Inhibition transition	Mean ± SEM (s) Forced off vs forced on LS: 1.9 ± 0.2 vs 1.8 ± 0.2; controls: 3.2 ± 0.3 vs 3 ± 0.3 Forced change rate (on–off) LS: −0.1 ± 0.1 vs controls: −0.1 ± 0.1	Paired two-sided permutation *t* test Unpaired two-sided permutation *t* test	0.45; 0.34 0.82	
	Breaks S1 Inhibition transition	Mean ± SEM (%) Forced off vs forced on LS: 0.6 ± 0.6 vs 5.5 ± 2.3; controls: 0 ± 0 vs 0.4 ± 0.4 Forced: change rate (on–off) LS: 4.8 ± 2.4 vs controls: 0.4 ± 0.4	Paired two-sided permutation *t* test Unpaired two-sided permutation *t* test	0.06; 1.0 0.01*	
	Breaks S2 Inhibition transition	Mean ± SEM (%) Forced off vs forced on LS: 16.8 ± 3.2 vs 15.9 ± 3.6; controls: 24.3 ± 3.9 vs 25.4 ± 4.4 Forced: change rate (on–off) LS: −0.8 ± 5 vs controls: 1 ± 4	Paired two-sided permutation *t* test Unpaired two-sided permutation *t* test	0.87; 0.82 0.80	
	Long-S1 sequences Inhibition transition	Mean ± SEM (%) Forced off vs forced on LS: 1.7 ± 1.7 vs 0.5 ± 0.5; controls: 3.5 ± 0.02 vs 6.2 ± 0.2 Forced: change rate (on–off) LS: −1.2 ± 1.9 vs controls: 2.7 ± 1.9	Paired two-sided permutation *t* test Unpaired two-sided permutation *t* test	0.50; 0.21 0.17	
	Transition Inhibition transition	Mean ± SEM (s) Forced off vs forced on LS: 0.8 ± 0.07 vs 0.9 ± 0.08; controls: 1.2 ± 0.1 vs 1.2 ± 0.1 Forced: change rate (on–off) LS: 0.1 ± 0.04 vs controls: −0.03 ± 0.02	Paired two-sided permutation *t* test Unpaired two-sided permutation *t* test	0.02*; 0.16 0.0006***	
	Presses Inhibition transition	Mean ± SEM (# presses) Forced off vs forced on LS: 7.7 ± 0.04 vs 7.5 ± 0.1; controls: 7.8 ± 0.1 vs 7.9 ± 0.1 Forced: change rate (on–off) LS: −0.1 ± 0.1 vs controls: 0.1 ± 0.1	Paired two-sided permutation *t* test Unpaired two-sided permutation *t* test	0.16; 0.41 0.18	
Data related to Figure 5	Breaks S1	Mean ± SEM (%) Forced off vs forced on M2-LS: 0 ± 0 vs 0 ± 0; M1-LS: 1.3 ± 0.9 vs 1.2 ± 0.8; controls: 0.8 ± 0.5 vs 0.8 ± 0.8 Forced: change rate (on–off) M2-LS: 0 ± 0 vs controls: 0.05 ± 0.6 M1-LS: −0.1 ± 1.1 vs controls: 0.05 ± 0.6	Paired two-sided permutation *t* test Unpaired two-sided permutation *t* test	1.0; 0.7; 0.5 0.5 0.9	Emx1-cre mice (+/−) AAV-Arch-M2-LS: *n* = 9 AAV-Arch-M1-LS: *n* = 8 AAV-eYFP-M2-LS: *n* = 9 (controls)
	Breaks S2	Mean ± SEM (%) Forced off vs forced on M2-LS: 15 ± 5 vs 16 ± 3; M1-LS: 18.9 ± 3.6 vs 15.9 ± 3.5; controls: 20 ± 3.8 vs 21 ± 5 Forced: change rate (on–off) M2-LS: 0.6 ± 4 vs controls: 0.6 ± 6 M1-LS: −3 ± 5 vs controls: 0.6 ± 6	Paired two-sided permutation *t* test Unpaired two-sided permutation *t* test	0.8;0.6;0.9 1.0 0.6	
	Sequence	Mean ± SEM (# presses) Forced off vs forced on M2-LS: 8.0 ± 0.2 vs 8.0 ± 0.1; M1-LS: 7.7 ± 0.1 vs 7.8 ± 0.1; controls: 7.7 ± 0.1 vs 7.6 ± 0.09 Forced: change rate (on–off) M2-LS: 0.07 ± 0.08 vs controls: −0.1 ± 0.1 M1-LS: 0.04 ± 0.9 vs controls: −0.1 ± 0.1	Paired two-sided permutation *t* test Unpaired two-sided permutation *t* test	0.43;0.67;0.33 0.19 0.29	
	Long-S1 sequences	Mean ± SEM (%) Forced off vs forced on M2-LS: 4.2 ± 3.6 vs 3.1 ± 2.3; M1-LS: 4.5 ± 1.9 vs 1.7 ± 1.2; controls: 2.6 ± 2.6 vs 2.6 ± 2 Forced: change rate (on–off) M2-LS: −1 ± 1.7 vs controls: 0.9 ± 0.02 M1-LS: −2.8 ± 1.2 vs controls: 0 ± 0.8	Paired two-sided permutation *t* test Unpaired two-sided permutation *t* test	0.5;1.0; 1.0 0.63 0.05	
	Duration	Mean ± SEM (s) Forced off vs forced on M2-LS: 5.19 ± 0.7 vs 4.7 ± 0.5; M1-LS: 4.1 ± 0.4 vs 4.22 ± 0.5; controls: 4.3 ± 1.0 vs 3.9 ± 0.6 Self-paced off vs self-paced on M2-LS: 6.1 ± 0.7 vs 7.3 ± 1.5; M1-LS: 6.0 ± 0.5 vs 6.0 ± 0.4; controls: 5.8 ± 1.2 vs 5.1 ± 0.9 Forced: change rate (on–off) M2-LS: −0.4 ± 0.4 vs controls: −0.4 ± 0.4 M1-LS: 0.03 ± 0.4 vs controls: −0.4 ± 0.5 Self-paced: change rate (on–off) M2-LS: 1.15 ± 1.2 vs controls: 1.0 ± 0.3 M1-LS: −0.05 ± 0.2 vs controls: 1.0 ± 0.3	Paired two-sided permutation *t* test Unpaired two-sided permutation *t* test	0.33;0.91;0.47 0.50;0.82;0.09 0.91;0.44 0.18;0.20	
Data related to Figure 6	Transition	Mean ± SEM (s) Forced off vs forced on M2-LS: 1.0 ± 0.1 vs 1.0 ± 0.1; M1-LS: 1.2 ± 0.2 vs 1.1 ± 0.1; controls: 0.9 ± 0.1 vs 0.9 ± 0.1 Forced: change rate (on–off) M2-LS: −0.04 ± 0.03 vs controls: 0 ± 0.02 M1-LS: −0.1 ± 0.14 vs controls: 0 ± 0.02	Paired two-sided permutation *t* test Unpaired two-sided permutation *t* test	0.21; 0.55; 0.8 0.34 0.59	Emx1-cre mice (+/−) AAV-Arch-M2-LS: *n* = 9 AAV-Arch-M1-LS: *n* = 8 AAV-eYFP-M2-LS: *n* = 9 (controls)
	Breaks S1	Mean ± SEM (%) Forced off vs forced on M2-LS: 0 ± 0 vs 0 ± 0; M1-LS:2.1 ± 1 vs 0 ± 0; controls:0 ± 0 vs 0 ± 0 Forced: change rate (on–off) M2-LS: 0 ± 0 vs controls: 0 ± 0 M1-LS: −2.1 ± 1 vs controls: 0 ± 0	Paired two-sided permutation *t* test Unpaired two-sided permutation *t* test	1.0; 1.0; 1.0 1.0 1.0	
	Breaks S2	Mean ± SEM (%) Forced off vs forced on M2-LS: 15 ± 4.6 vs 18 ± 4.4; M1-LS: 18 ± 4.8 vs 12 ± 3; controls: 24 ± 5 vs 22 ± 3 Forced: change rate (on–off) M2-LS: 2.7 ± 4 vs controls: −1.7 ± 5 M1-LS: −5.9 ± 4 vs controls: −1.7 ± 5	Paired two-sided permutation *t* test Unpaired two-sided permutation *t* test	0.49;0.24;0.70 0.48 0.55	
	Duration	Mean ± SEM (s) Forced off vs forced on M2-LS: 4.8 ± 0.3 vs 4.1 ± 0.3; M1-LS: 3.8 ± 0.4 vs 3.9 ± 0.4; controls: 3.4 ± 0.4 vs 3.8 ± 0.4 Self-paced off vs self-paced on M2-LS: 5.3 ± 0.5 vs 7.7 ± 1.6; M1-LS: 6.2 ± 0.6 vs 6.1 ± 0.5; controls: 4.8 ± 0.6 vs 4.5 ± 0.5 Forced: change rate (on–off) M2-LS: −0.7 ± 0.4 vs controls: 0.3 ± 0.2 M1-LS: 0.08 ± 0.1 vs controls: 0.3 ± 0.2 Self-paced: change rate (on–off) M2-LS: 2.4 ± 1.7 vs controls: −0.2 ± 0.1 M1-LS: −0.08 ± 0.2 vs controls: −0.2 ± 0.1	Paired two-sided permutation *t* test Unpaired two-sided permutation *t* test	0.12;0.46;0.07 0.23;0.69;0.08 0.04*;0.52 0.14;0.57	
	# Presses sequences	Mean ± SEM (# presses) Forced off vs forced on M2-LS: 7.9 ± 0.2 vs 7.7 ± 0.07; M1-LS: 7.7 ± 0.07 vs 7.8 ± 0.05; controls: 7.8 ± 0.2 vs 8.0 ± 0.2 Self-paced off vs self-paced on M2-LS: 10.9 ± 0.6 vs 11.9 ± 0.7; M1-LS: 11.8 ± 0.7 vs 11.8 ± 0.5; controls: 11.1 ± 0.3 vs 10.7 ± 0.4 Forced: change rate (on–off) M2-LS: −0.2 ± 0.1 vs controls: 0.1 ± 0.1 M1-LS: 0.05 ± 0.08 vs controls: 0.1 ± 0.1 Self-paced: change rate (on–off) M2-LS: 1.0 ± 0.8 vs controls: −0.3 ± 0.3 M1-LS: 0.01 ± 0.2 vs controls: −0.3 ± 0.3	Paired two-sided permutation *t* test Unpaired two-sided permutation *t* test	0.14;0.67;0.19 0.27;0.97;0.25 0.049*;0.55 0.11;0.37	

For the first exposure to the operant box, animals were placed in the box without levers for 30 min. A total of 30 pellets were delivered individually at random intervals (on average every 60 s). Over the next 3 d, the animals were presented with lever 1 or lever 2 and received a reward each time they pressed the lever. After eight rewards, lever 1 was retracted, and lever 2 was presented. The session finished once the animal got 16 pellets, or 30 min had passed. Afterward, the training schedule changed to 3 d at a fixed ratio eight (FR8) schedule on each lever, whereby animals had to accumulate eight lever presses to receive a reward and retract the lever, followed by a second lever presentation, which also required 8 presses to receive the reward and retract the second lever (pretraining). The session finished when mice reached 30 rewards (15 on lever 1, 15 on lever 2) or 30 min had passed. If the animals checked the magazine before reaching eight continuous lever presses, a time out (10 s) was presented. We used 3 d in FR8 since in previous studies have seen this induces animals to press in bouts of around four presses (Tecuapetla et al., 2016). Next, animals were trained to do forced serial order sequences (3 d). Here, animals were presented with a lever 1; they had to do four continuous lever presses for the lever to be retracted, followed by extension of a second lever (lever 2). Animals had to do four continuous lever presses on lever 2 for it to be retracted, and a reward was delivered into the magazine (Fig. 1*B*). If the animals executed fewer than four lever presses on lever 1 or lever 2 by visiting the magazine, a time out (10 s) was presented. The session finished when mice got 30 rewards, or 30 min had passed. After these 3 d of forced sequences, the animals entered the last stage of training: blocks of forced–self-paced sequence sessions. These sessions began with a block of forced trials, switching to a block of self-paced trials in the same session. The switch between blocks was conditioned by achieving five consecutive correct sequences. For the self-paced sequences, the animals were presented with the two levers at the same time. They had to do at least four presses on lever 1 followed by at least four presses on lever 2, to obtain a reward in the magazine. Unlike in forced blocks, in self-paced blocks the animals decided when to execute the transition between the subsequences of presses from lever 1 to lever 2 (Fig. 1*C*). Upon finishing a self-paced block, both levers are retracted for 3 s for an intertrial interval before starting a new forced block. If the animals executed fewer than four presses on lever 1 or lever 2 by visiting the magazine, a time out (10 s) was presented. The session finished once the animals received 70 rewards (35 forced and 35 self-paced rewards) or 30 min had passed. The block sessions continued for 11–13 d until a stable performance was reached; see Fig. 1*E*). All timestamps were recorded with a resolution of 10 ms with the Med-PC IV software suite (Med-Associates).

### Single-unit recording and antidromic photo-identification

To record the cortical activity, either a fixed or movable 16-electrode array [tungsten (35 μm; Innovative Neurophysiology) was implanted]. The neurons’ spikes were sorted online (Central software, Blackrock Microsystems), and clear waveforms with a signal-to-noise ratio >3:1 were used for further analysis using offline-sorting (Offline Sorter, Plexon Inc.). To define whether a recorded unit projected to the striatum, we used *in vivo* antidromic photo-identification (Lima et al., 2009). In short, we injected ChR2 (UPENN-vector core catalog #AV-1-20298P) in the cortex of interest of Emx1-Cre animals. A movable electrode array was implanted 200 μm above the ChR2 expression site during the same surgery, and an optic fiber (Thorlabs catalog #CFLC230-10, FT200EMT) was implanted into the striatum (ipsilateral to injection). Using the electrode array, we could record activity of cortical neurons. At the end of the behavior session, we used a light stimulation (2 mW, 473 nm, 10 Hz, 1 s, 10-ms pulses; Laserglow) delivered by the optic fiber while recording neuronal activity. This allowed us to verify whether the recorded neuron was antidromically photo-activated (Extended Data Fig. 3-2). The movable array allowed us to search for responding cells in at least five sessions per animal, advancing the array 100 μm 24 h before the recording. Only units that responded to the light (i.e., presented a correlation of >0.9 between the behavioral spike and the antidromic spike and presented a latency to light <10 ms) were considered.

### Per-trial rescaling of neural activity

In both sequence types, forced and self-paced, each sequence had a slightly different durations and could not be directly averaged. To mitigate this, we employed a time rescaling procedure (Kobak et al., 2016), to evaluate each recorded unit’s overall response pattern. Thus, we performed a rescaling of trials with at least four presses in each sequence, defining the following alignment events: first, second, penultimate, and last press of subsequence 1 and subsequence 2. Spike trains were transformed to instantaneous firing rate by applying a Gaussian convolution (σ 25 ms) at 100 Hz. The activity was rescaled through linear interpolation to the average interpress interval and inter sequence interval of all recorded animals’ sessions.

### Analysis of the electrophysiological recordings *in vivo*

Once a putative unit was isolated (through online and off-line sorting), the spikes’ timestamps were aligned to the first lever press in the sequences of actions using custom-developed scripts in MATLAB (MathWorks).

**Figure 2.**
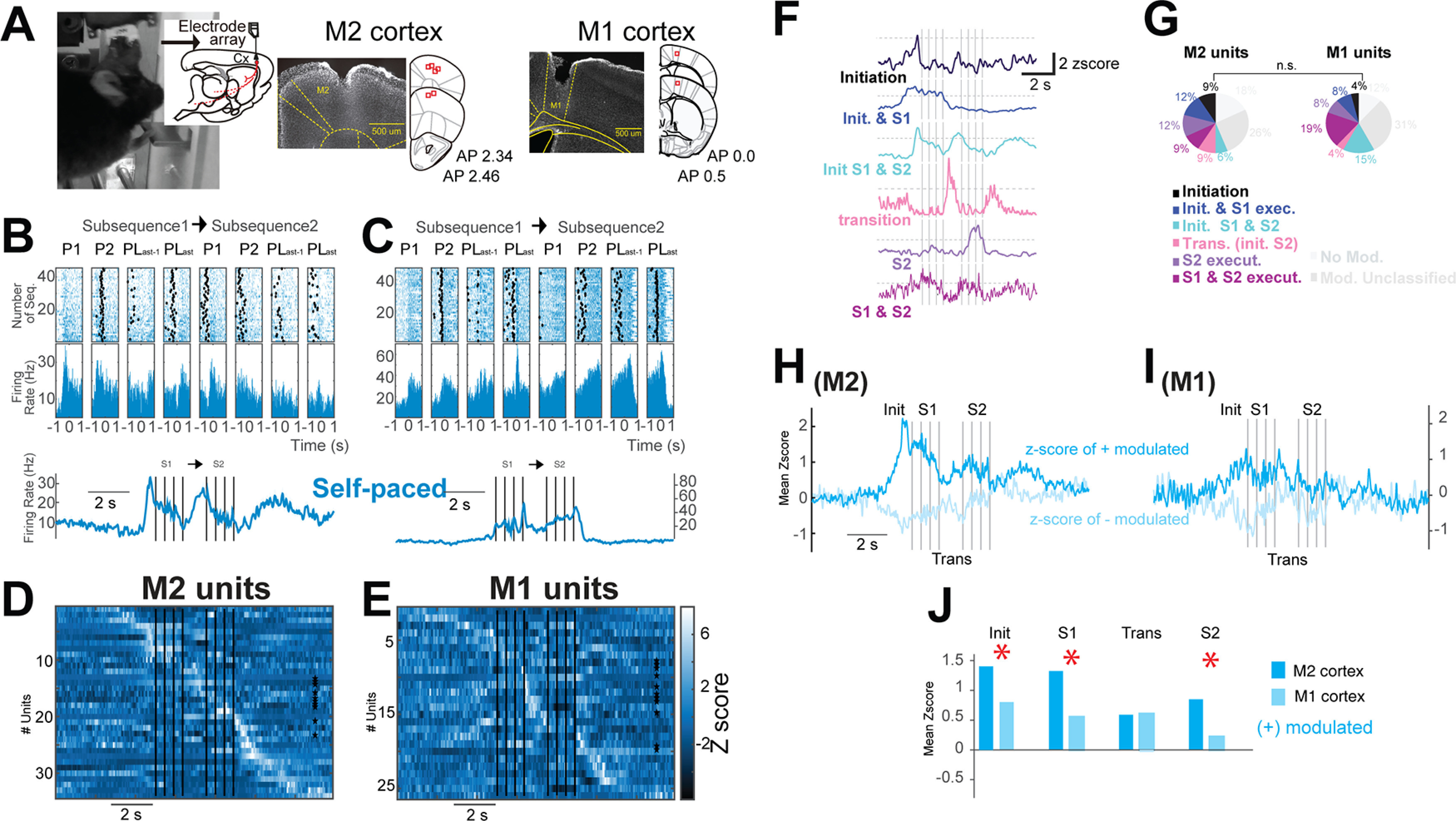
Activity modulation in premotor and motor cortical neurons during the execution of self-paced sequences of actions. ***A***, Image of a mouse while pressing one of the levers and photomicrographs of coronal sections of M2 (middle) or M1 cortex (right), illustrating the cannula/electrode array’s tracks in the recorded sites. ***B***, ***C***, Raster plots and perievent histogram from an M2 or an M1 unit, respectively, aligned to the first (P1), second (P2), penultimate (PLast-1), and last lever press (PLast) of S1 and S2 for self-paced sequences. Bottom panels, Mean firing rate from the upper panels. ***D***, ***E***, *Z* score of individual units. ***F***, Representative firing of different cells presented as *z* score, illustrating the different categories of modulation during the execution of the sequences Init & S1 execu = Initiation and S1 execution; Init. S1 & S2 = Initiation of S1 and S2; Trans.(init. S2) = Transition or initiation S2; S2 execut. = S2 execution; S1 & S2 execut. = S1 and S2 execution. ***G***, % of cells related to each category in ***F***. ***H***, ***I***, Mean *z* score from the units recorded that presented significant modulation 1 s before the initiation of the sequence from M2 or M1. ***J***, Comparison of the mean *z* score from ***H***, ***I***, M2 (dark blue) versus M1 (light blue) for positively modulated units; *p < 0.05, *z* score test. Init. = initiation, S1 = subsequence 1, Trans = transition, and S2 = subsequence 2. Extended Data Figure 2-1 includes the activity modulation in premotor and motor cortical neurons during the execution of forced sequences of actions. n.s. = *p* > 0.05.

*Z* score test: to determine differences between the *z* score activity from M2 versus M1, we averaged the *z* score activity in the same time window from the two indicated cells and calculated the difference in terms of *z* score with the following equation:

$$Zdifference=\frac{Z1-Z2}{\sqrt{\left(\frac{1}{N1-3}\right)+\left(\frac{1}{N2+3}\right)}},$$

where Z1 is the average normalized activity of the M2 units and Z2 for the M1; N1 is the sample of units from M2, and N2 the sample of units from M1 (Fig. 2*J*; Extended Data Fig. 2-1*H*). The results were compared in a normal distribution table to determine the corresponding *p* value (Chen and Popovich, 2011).

10.1523/ENEURO.0173-21.2021.f2-1 Extended Data Figure 2-1 Activity modulation in premotor and motor cortical neurons during the execution of forced sequences of actions. ***A***, ***B***, Raster plots and perievent histogram from an M2 or an M1 unit, respectively, aligned to the first (P1), second (P2), penultimate (PLast-1), and last lever press (PLast) of S1 and S2 for forced sequences. Bottom panels, Mean firing rate from the upper panels. ***C***, ***D***, *Z* score of individual units. ***F***, ***G***, Mean *z* score from the units recorded that presented significant modulation 1 s before the initiation of the sequence from M2 or M1. ***H***, Comparison of the mean *z* score from ***F***, ***G***, M2 (dark red) versus M1 (light red) for positively modulated units. ***I***, Percentage of recruited units (bins of 200 ms, a sliding window of 10 ms) for M2 versus M1 during forced and self-paced sequences; *p < 0.05, *z* score test. Init. = initiation, S1 = subsequence 1, Trans = transition, S2 = subsequence 2. Download Figure 2-1, TIF file.

### Regression analysis of the neuronal activity and behavior

**Figure 3.**
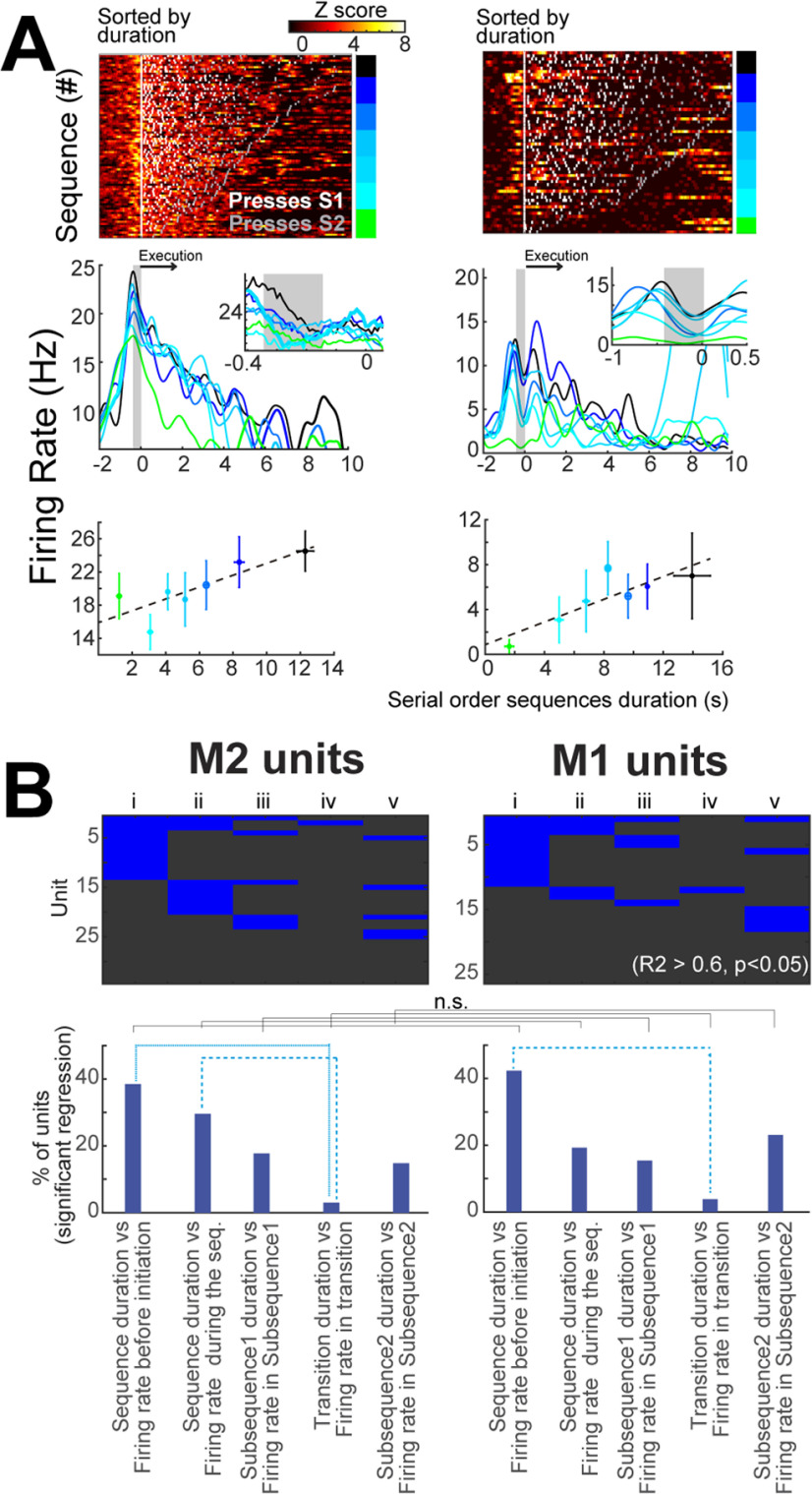
Both M2 and M1 contain units encoding the temporality of the self-paced sequences of actions. ***A***, top panels, Examples of two units recorded in M2. In each example, each line is a self-paced sequence depicting the neuronal activity (in *z* score, black to yellow) and lever press (white points subsequence 1, gray points subsequence 2). The color bar to the right of each plot shows the grouped categories plotted in the middle and bottom panels. Middle row panels, Mean firing rate from each category presented in the upper row. Bottom row, Regression fits from the time bin depicted in gray in the middle row panels. ***B***, top row panels, Significant regression analysis per unit; columns i, between the firing rate (FR) 1 s before the start of the sequences (Seq) versus the duration of the sequences; columns ii, FR during the sequences versus duration of sequences; columns iii, FR in the subsequence 1 (S1) versus the duration of S1; columns vi, FR during the transition versus the transition time; and columns v, FR during the subsequence 2 (S2) versus the S2 duration. Bottom panels, The proportion of units that presented significant regression (*R*^2^ > 0.6 and *p* < 0.05). The dashed lines depict comparisons with χ^2^ test. Corrections for multiple comparison was considered (see Materials and Methods). Extended Data Figure 3-1 shows the recorded units in M2 or M1 encoding the temporality of the forced sequences of actions. Extended Data Figure 3-2 shows the confirmation of the M2-M1 projections into the LS and linear regressions between the activity and the temporal parameters of the execution of sequences from the photo-identified M2 or M1 cortico-striatal neurons. n.s. = *p* > 0.05.

We made linear regression analysis to ask whether the firing rate in each bin of time was correlated with parameters of the task. For this purpose, we first aligned the spikes to each epoch (first, second, penultimate, and last lever press for each sequence) and took 5 s before and 5 s after each epoch. Spike trains were transformed to instantaneous firing rate as described above. We calculated the spike frequency using a 200-ms time window with 10-ms steps. We separated the trials and sorted them by the number of lever presses in the sequences (2,4,6, up to 16, grouped by every increment), for the sequence and transition duration (sorted by duration in descending manner, seven categories), for latency (grouped every one second, starting with 0.5 s.). Then we made a regression analysis with permutation test in two manners. For the first, we used a bin of time of 200 ms with a sliding window of 10 ms and ask whether the variable of interest was correlated with the firing rate (Fig. 3*A*; Extended Data Fig. 3-1*B*). For the second regressions analysis, we used specific windows of time: (1) mean firing rate 1 s before the start of the sequence; (2) mean of firing rate during the sequence; (3, 4, and 5) average of firing rate during S1, firing rate during transition or firing rate during S2 (Fig. 3*B*). To resolve statistically whether the regression’s *p* value was significant, we ran 1000 permutations and divided the sum of times that the *p* value > *p* value initial between the number of permutations. Only regressions with β coefficient different from zero (*p* < 0.05 and *R* > 0.6) were accepted.

10.1523/ENEURO.0173-21.2021.f3-1 Extended Data Figure 3-1 Both M2 and M1 contain units encoding the temporality of the forced sequences of actions. ***A***, top row panels, Significant regression analysis per unit columns i, between the firing rate (FR) 1 s before the start of the sequences (Seq) versus the duration of the sequences, columns ii: FR during the sequences versus duration of sequences, columns iii: FR in the subsequence 1 (S1) versus the duration of S1, columns vi: FR during the transition versus the transition time, and columns v: FR during the subsequence 2 (S2) versus the S2 duration. Bottom panels, The proportion of units that presented significant regression (*R*^2^ > 0.6 and *p* < 0.05). The dashed lines depict comparisons with χ^2^ test. Corrections for multiple comparison was considered (see Materials and Methods). ***B***, Percentage of recruited units (presenting significant regression, bins of 200-ms sliding window of 10 ms regarding the tittles in each plot). Bars show the average of the recruited units 1 s before and after the start of the sequences except for the transitions, which is aligned to the last press of the subsequence 1. Download Figure 3-1, TIF file.

10.1523/ENEURO.0173-21.2021.f3-2 Extended Data Figure 3-2 Confirmation of the M2-M1 cells and their projections into the LS and linear regressions between the activity and the temporal parameters of the execution of sequences from the photo-identified M2 or M1 cortico-striatal neurons. ***A***, Scheme of retrograde tracing of M2 and M1 neurons (left) labeled by retrobead injection into the LS (right). ***B***, left panels, Anteroposterior coronal photomicrographs showing retrobead labeled cells in M2/M1 (left) from the retrobead injections into the LS. Scale bar: 500 μm. Right panels, Diagrams presenting summed data from four animals that were injected with retrobeads into the LS in binary color code. ***C***, Scheme of premotor cortex anterograde tracing using AAV-Retro-Cre-mCherry injection into the LS (injection1) and AAV-DIO-eYFP into the premotor cortex (M2; injection2). ***D***, Left photomicrographs depict an example from one animal of the experiment described in ***C***. Prelimbic cortex (PL), cingulate cortex (Cg1). Scale bar: 500 μm. Right top panels, Zoom-in of the LS and the medial striatum (MS) showing the premotor cortico-striatal projections labeled in green. Scale bar: 50 μm. The bars show the quantification of the M2→LS fibers reaching the LS at AP. 0.3 from bregma (coordinate where most of the fiber tips of the optogenetic inhibition were found). ***E***, Diagram of the injection site to express ChR2 into M1 or M2 cortex and their projections, the electrode array (M2 or M1 cortex), and the optical fiber implantation (LS). ***F***, Raster-plot example of a cortico-striatal PID unit and the perievent time histogram aligned at the start of the first pulse of a train of blue light (10 Hz; 2 mW, 473 nm). Right panels, Mean waveforms, principal component analysis, and latency to response to stimulation during behavior (black) and photo-identification protocol (blue). ***G***, top row panels, Significant regression analysis per unit; columns i, between the firing rate (FR) 1 s before the start of the sequences versus the sequences duration; columns ii, FR during the sequences versus sequences duration; columns iii, FR in the subsequence 1 (S1) versus the duration of S1; columns vi, FR during the transition versus the transition time; and columns v, FR during the subsequence 2 versus the subsequence 2 duration. Bottom row panels, Proportion of units that presented significant regression (*R*^2^ > 0.6 and p < 0.05) during forced (red) or self-paced sequences (blue). Despite tendencies, no significant differences were found in the comparisons within or between cortical regions, χ^2^ test. Download Figure 3-2, TIF file.

### ROC curve analyses and permutations

To determine the percentage of modulated units along time in each epoch, we performed a ROC curve analysis to ask whether the spike frequency in each time bin was different from baseline time. We first aligned the spikes to each epoch (e.g., first press) using 4 s before and 5 s after each epoch. We calculated the spike frequency using a 200-ms time window. We used a baseline from –4.5 to –4 s before the first press in the sequences. We compared the spike frequency in each bin of time against the baseline, using a sliding window of 10 ms. To resolve statistically whether the area under the curve (AUROC) was significant, we ran 1000 permutations and divided the sum of AUROC values that fall in either >0.5 or <0.5, by the number of permutations. The AUROC value was significant if the outcome was *p* < 0.05. Furthermore, in each epoch, we obtained a binary matrix comparing each bin to baseline. This binary matrix was used to find the number of units modulated in each time bin. For the linear regression, to resolve statistically whether the regression’s *p* value was significant, we ran 1000 permutations and divided the sum of times that the *p* value > *p* value initial between the number of permutations.

### Stereotaxic opsin injection and fiber implantation

For surgeries, animals were anesthetized using a mix of oxygen (1 l/min) and 1% isofluorane (1–2% for interventional procedures). For the optogenetic experiments: after anesthesia, each animal was bilaterally injected using glass pipettes with 500 nl of viral stock solution [either rAAV5-EF1a-DIO-eArch3.0-EYFP (Vector core, University of North Carolina), or AAV1.EF1a.DIO.eYFP.WPRE; AAV1.EF1a.DIO.hChR2(H134R)-eYFP.WPRE titer > 1 × 1012 (Vector core UPENN University Pennsylvania catalog #AV-127056)] by pressure into either the M2 or M1 or lateral striatum (LS), coordinates from bregma, M2: AP 2.34 mm, ML 1.25 mm, DV 0.60–0.70 mm. M1: AP 0.5 mm, ML 1.60 mm, DV 0.60–0.70 mm. LS: AP 0.50 mm, ML 2.50 mm, DV 2.40 mm below the brain’s surface. After the injections were done (23 nl every 5 s; Nanoject II, Drummond Scientific), we waited 15 min to allow time for virus to spread, and a fiber-optic (300 μm; NA 0.37) was implanted into each hemisphere of the striatum. The optical fibers were fixed to the skull using acrylic cement (Lang Dental Manufacturing Co, Inc).

### Retrobead injections

For the retrograde labeling experiments, 300 nl of retrobeads (Lumafluor) were injected into the LS. Coronal sections (50 μm) were obtained to determine the total number of cells labeled in the M2 or M1. The quantification was done in one slice every 300 μm covering these regions.

### Retro-Cre injections

Similarly, to the retrobead injections, 300 nl of mCherry-Retro-Cre (Addgene catalog #55632-AAVrg, RRID: Addgene_55632) AAV were injected into the LS, and 300 nl of DIO-eYFP were injected into the M2 cortex of Emx1-Cre. Coronal striatal sections of 50 μm were obtained to determine the axons crossing by the dorsomedial or the dorsolateral striatum.

### Cortico-striatal fibers quantification

The axonal quantification protocol was as previously reported (Díaz-Hernández et al., 2018). In short, we extracted the brains and sectioned the striatum (50-μm coronal sections). Z stacks at 63× magnification were acquired (192 × 192 × 10 μm; 1-μm interslice) from a random quadrant; using a randomly positioned grid covering either the dorsolateral or the dorsomedial striatum (ZEN lite software, Zeiss, LSM 710). These Z stacks were imported into ImageJ; then, a maximum projection image was used to apply a filter (Hessian filter), allowing the quantification of fibers as defined by the number of fibers crossing a randomly generated line spaced ∼20 μm.

### Cortical microstimulation of forelimb region in the M1

Micro-stimulation experiments were performed to identify the M1’s coordinates corresponding to the contralateral forelimb region. The animal was placed in the stereotaxic apparatus under anesthesia (ketamine 0.15 mg/g mouse + xylazine 0.01 mg/g mouse). Access to motor cortex was achieved by trepanning a window of at least 1 mm in diameter around the center point (AP +1, ML 1). Electrical stimulation was performed using a 300-μm concentric bipolar electrode on the dura’s surface with 15 square pulses of 200 μs at a frequency of 200 Hz using a stimulator device (DS2, Digimiter). With the administration of voltage pulses, we looked for contralateral forepaw movement and corroborated this using a camera. This stimulation was performed on a grid every 100 μm in the AP and ML direction. The coordinates where the stimulation resulted in movement of the contralateral brachial biceps were taken to implant the recording electrode (mouse1: AP 0.5, ML 1.5; mouse 2, AP 0.0, ML 1.5).

### Temporally defined optogenetic striatal inhibition *in vivo*

**Figure 4.**
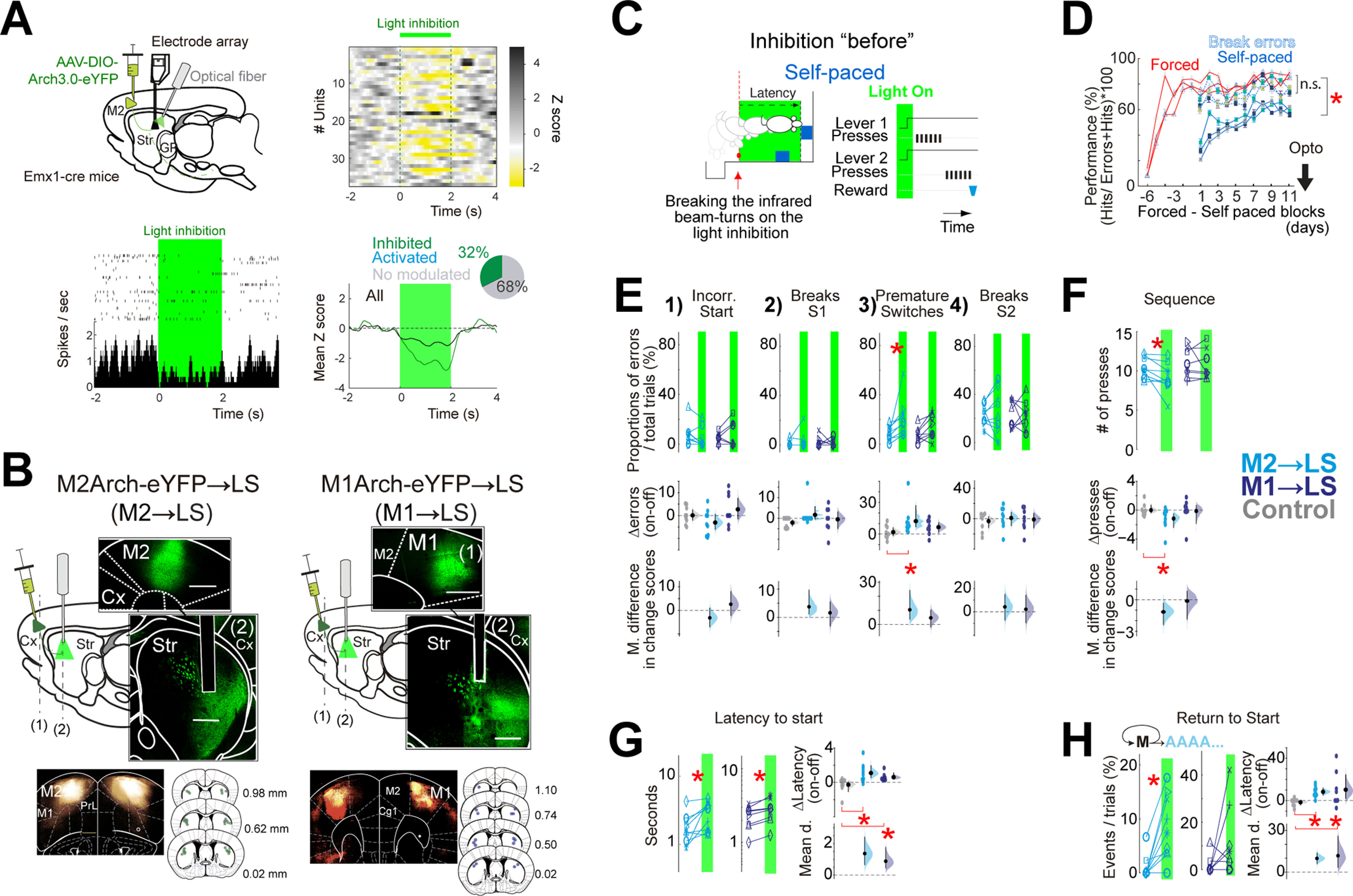
The inhibition of the premotor and primary motor cortico-striatal projections before sequence initiation impairs the initiation, but only the premotor disrupts the execution. ***A***, top left, Diagram illustrates the injection site of Arch3.0-eYFP and the optrode (electrode array+ fiber optic) implantation into the LS. Top right, Plot depicting the activity of several units recorded in the striatum (*z* score) aligned to the inhibition of the M2 cortico-striatal projections into the LS (green line above). Bottom left, representative perievent time histogram and raster plot of a striatal unit’s activity aligned to the inhibition of the M2 cortico-striatal projections (green shadow). Bottom right, Mean *z* score for the units that decreased their activity (green) or for all units recorded (black) during the inhibition. Inset, Pie chart showing the proportion of modulated and non-modulated units (comparing baseline time vs light). ***B***, top panels, Sagittal diagrams and cortical photomicrographs of the injection of Arch3.0-eYFP into either M2 (*n* = 9) or M1 (*n* = 8) cortices and optical fibers implantation into the LS (Str). Scale bar: 500 μm. Bottom panels, Photomicrograph showing the average of Arch3.0-eYFP expression of the corresponding groups. Coronal diagrams representing the position of the optical fiber’s tips into the LS (green dots: Arch3.0-eYFP; gray dots: eYFP). ***C***, Scheme of the inhibition protocol before the initiation of the sequences. The light inhibition (2 s) is triggered by breaking the infrared beam positioned outside of the magazine, when coming to the lever (dashed red lines on the schemes). ***D***, Percentage of correct sequences [correct/(errors + correct)] throughout training. ***E***, upper panels 1–4, Quantification per animal in off versus on trials of the proportion of each category of error. ***F***, Effect of the inhibition on the number of lever press per sequence. ***G***, Effect of the inhibition of the cortico-striatal projections on the latency to initiate self-paced sequences. ***H***, Effect of the inhibition of the cortico-striatal projections on the proportions of times per block that animals returned to the magazine after having crossed the infrared sensor outside of the magazine (which set the timestamp to trigger the light inhibition; see Movie 3). On the paired plots, each line depicts the mean effect per animal during trials of optogenetic inhibition (on; green shadow) versus trials without optogenetic inhibition (off; no shadow) from the same session. In panels ***E–H***, Δ on-off panels are obtained from the mean difference per animal in the on-off trials, adding the control group in gray. The mean difference in change score panels (mean d.) are obtained between the experimental groups and the control group (see Materials and Methods). Paired plots, two-sided permutation *t* test; panels Δ on-off, Arch animals versus control eYFP animals, unpaired two-sided permutation *t* test; *p < 0.05. The exact *p* values are described in the text and Table 1. Extended Data Figure 4-1 shows the effects of the inhibition of the lateral striatal neurons during the initiation and execution of the action sequences. Extended Data Figure 4-2 shows the M2/M1 retrogradely labeled cells from the LS, the thalamus, and the pons. Extended Data Figure 4-3 shows that the premotor cortico-striatal projections innervate the direct pathway with a stronger synaptic weight than the indirect pathway. n.s. = *p* > 0.05.

**Figure 5.**
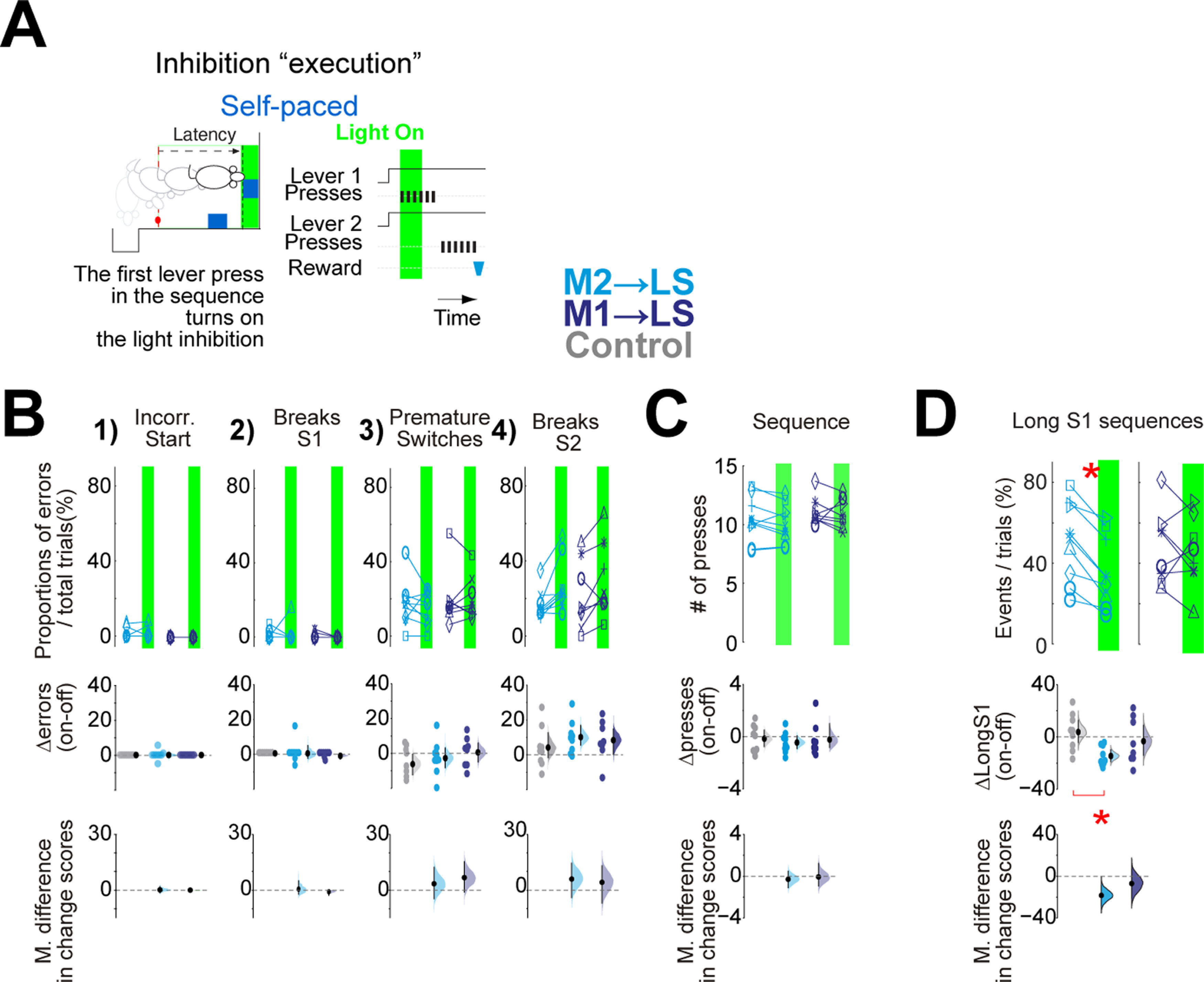
The inhibition of premotor cortico-striatal projections at the start of the execution decreased Long-S1 self-paced sequences. ***A***, Scheme of the inhibition execution protocol. Light inhibition was triggered by the first press in the sequences. The green shadow depicts 2 s of continuous light inhibition. ***B***, upper panels 1–4, Quantification per animal in off versus on trials of the proportion of each category of error. ***C***, Effect of the inhibition on the number of lever presses per sequence. ***D***, As in ***B***, ***C*** evaluating the proportion of animals performing Long-S1 sequences S1 > 4→S2 >= 4). On the paired plots, each line depicts the mean effect per animal during trials of optogenetic inhibition (on; green shadow) versus trials without optogenetic inhibition (off; no shadow) from the same session. In panels ***B–D***, Δ on-off panels are obtained from the mean difference per animal in the on-off trials, adding the control group in gray. The mean difference in change score panels (mean d.) are obtained between the experimental groups and the control group (see Materials and Methods). Paired plots, two-sided permutation *t* test; panels Δ on-off, Arch animals versus control eYFP animals, unpaired two-sided permutation *t* test; *p < 0.05. The exact *p* values are described in the text and Table 1.

**Figure 6.**
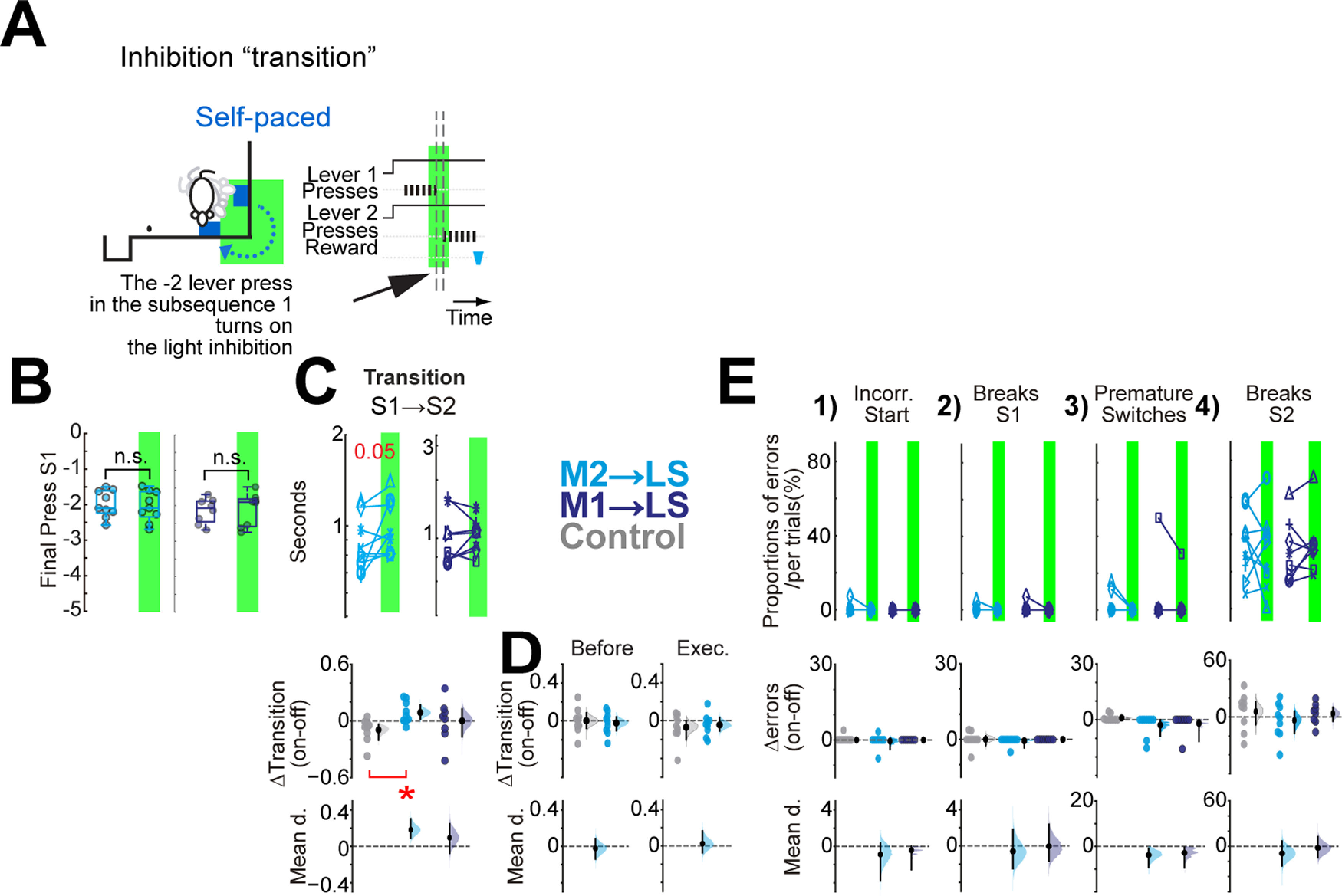
Inhibition of premotor but not primary motor cortico-striatal projections at the moment of the transition increases the transition time inside self-paced sequences. ***A***, Scheme of the inhibition protocol transition in self-paced sequences. The light inhibition was triggered by the press before the penultimate press of the subsequence 1. The dashed and black arrows denote the period of the transition. ***B***, Estimation of the press that delivered the timestamp for light inhibition for M2→LS and M1→LS, light and dark blue, respectively. ***C***, upper panels, Effect of the inhibition of the premotor or primary motor cortico-striatal projection on the transition time for self-paced sequences. ***D***, Δ on-off for the inhibition before and execution protocols from the inhibition of M2→LS presented in Figures 4, 5, respectively. ***E***, No effect was detected by the inhibition of either M2→LS or M1→LS projection in the different categories of errors. Upper panels 1–4, Quantification per animal in off versus on trials of the proportion of each category of error. In ***C***, ***E***, each line in the paired plots depicts the mean effect per animal during trials of optogenetic inhibition (on; green shadow) versus trials without optogenetic inhibition (off; no shadow) from the same session. The panels Δ on-off are obtained from the mean difference per animal in the on-off trials, adding the control group in gray. The mean difference in change score panels (mean d.) are obtained between the experimental groups and the control group (see Materials and Methods). Paired plots, two-sided permutation *t* test; panels Δ on-off, Arch animals versus control eYFP animals, unpaired two-sided permutation *t* test; *p < 0.05. The exact *p* values are described in the text and Table 1.

Light was delivered via 300 micrometer-diameter implantable fibers (Doric lenses) coupled to a single longitudinal mode laser (MSL-FN-556, CNI lasers; 556 nm). For the optogenetic inhibition experiments, a free launching system controlled by an AOM (AAoptoelectronics) and fast speed shutter (Thorlabs catalog #SH5, SC10) triggered by TTL output from the MED-PC behavioral box was used to deliver the light. Power at the fiber tip was verified for every experiment using a power meter (Thorlabs catalog #PM130D). The power was adjusted to be 20 mW at the tip of the fiber for the green light. To define the time point for the optogenetic inhibition of the cortico-striatal projections before the initiation of sequences, we took advantage of the fact that animals developed stereotypical sequences. Thus, when the animal moved from the magazine to the first lever (left lever), the infrared beam was broken, sending the timestamp to trigger the light on and allowing the quantification of the latency to initiate the sequences of actions (Figs. 1*B*,*C*, 4*G*; Extended Data Fig. 4-1*C*,*I*,*O*). To define the time point for the light manipulations during the sequences’ execution, we used the timestamp of the first lever press in the sequences (Fig. 5*A*). To define the time point for the light manipulations during the transition between forced subsequences, we used the timestamp of the penultimate lever press of subsequence 1. In self-paced sequences, we quantified the mean of lever presses to define a penultimate lever press (although we confirmed that it was two presses before the last of S1 in this case; see Fig. 6*B*). During the session of optogenetic inhibition, there were control (light off) trials and stimulation trials. The stimulation trials were randomly presented throughout the session (50% of total trials).

10.1523/ENEURO.0173-21.2021.f4-1 Extended Data Figure 4-1 Inhibition of the lateral striatal neurons impairs the initiation and execution of sequences of actions. ***A***, panel 1, Diagram of the injection of archaerhodopsin 3.0-eYFP (under the synapsin promoter; Syn-Arch3.0) into the LS and the corresponding site for fiber optic implantation. The photomicrograph shows a coronal section at the striatal level depicting the Arch3.0-eYFP expression of a single animal. Panel 2, Location of the fiber optic tips into the LS from the eight animals considered for this group (green points). The coronal section with the shadows in grey represents the extension of the Arch3.0 expression. Panel 3, Percentage of correct sequences [correct/(errors + correct)] along with the training. ***B***, ***H***, ***N***, Schemes of the inhibition protocols applied in the corresponding columns. Green shadows depict 2 s of continuous light, forced (red), and self-paced sequences (blue). ***B***, Inhibition before the initiation, triggered by the breaking of an infrared bean placed outside of the magazine towards the lever press. ***H***, Inhibition triggered by the first press in the execution. ***N***, Inhibition triggered by the penultimate press in the subsequence 1 of the sequences. ***C***, ***I***, ***O***, Effect of inhibition of lateral striatal neurons on the latency to initiate forced or self-paced sequences. ***D***, ***J***, ***P***, ***E***, upper panels 1–4, Quantification per animal in off versus on trials of the proportion of each category of error. ***E***, ***K***, ***Q***, As in ***C***, ***I***, ***O*** evaluating the proportion of Long-S1 sequences (S1 > 4→S2 >= 4). ***F***, ***L***, ***R***, As in ***C***, ***I***, ***O*** evaluating the transition time in forced and self-paced sequences. ***G***, ***M***, ***S***, As in ***C***, ***I***, ***O*** evaluating the number of presses in forced and self-paced sequences. In each paired graph, each line plots the mean effect per animal during trials of optogenetic inhibition (on; green shadow) versus trials without inhibition (off; no shadow) from the same session. In panels ***C–S***, Δ on-off panels are obtained from the mean difference per animal in the on-off trials, adding the control group in grey. The mean difference in change score panels (M. d.) are obtained between the experimental groups and the control group (see Materials and Methods). Paired plots, two-sided permutation *t* test; panels Δ on-off, Arch animals versus control eYFP animals, unpaired two-sided permutation *t* test; *p < 0.05. The exact *p* values are described in the text and Table 1. Download Figure 4-1, TIF file.

10.1523/ENEURO.0173-21.2021.f4-2 Extended Data Figure 4-2 M2/M1 retrogradely labeled cells from the LS, the thalamus (Th), and the pons (Pns). ***A***, Diagram of the experiment to trace corticofugal projections to the thalamus or to the pons leaving collaterals/crossing by the LS. Top left, First retrograde tracer injection (green retrobeads) into the LS. Bottom panels, Second retrograde tracer injection (red retrobeads) into the thalamus or the pons. Top right, Diagram of a sagittal section showing the anteroposterior level of the striatum, the thalamus or the pons (black vertical lines). Bottom right, A coronal diagram illustrating the M2 cortical projection to the LS (green), the thalamus (red), or both (yellow). ***B***, Photomicrographs of the sites of injection of retrobeads into the LS (green) or the thalamus (Th; red) or the pons (Pns; red). ***C***, left panels, green, M2/M1 cortico-striatal cells; red, M2/M1 cortico-thalamic cells. Right panels, Zoom-in from the pink squares on the left. ***D***, Percentage of single target cells and cells reaching either the thalamus or the pons leaving collaterals/crossing at the LS. Green, The proportion of cells only targeting the LS; red, the proportion of cells targeting either the thalamus (top) or the pons (bottom); yellow, the proportion of cells that targeted the LS and the thalamus (top) or the LS and the pons (bottom) simultaneously. Download Figure 4-2, TIF file.

10.1523/ENEURO.0173-21.2021.f4-3 Extended Data Figure 4-3 Premotor cortico-striatal projections innervate the direct pathway with a stronger synaptic weight than the indirect pathway. ***A***, Diagram of *ex vivo* experiments to record the cortico-striatal synaptic connectivity on the two kinds of striatal projection neurons, striatopallidal (iMSN) or striatonigral (dMSN). (1) Coronal diagram showing the injection of ChR2 into premotor cortex (M2). (2) Coronal diagram showing the premotor cortex projections onto iMSN (green dots) or dMSN (red dots). (3) Coronal diagram showing the injection of retrobeads into the SNr. ***B***, left, Photomicrograph of ChR2 injection into M2. Middle, A photomicrograph of the lateral part of the striatum showing the premotor cortex projections (eYFP; green) and the dMSN labeled with retrobeads (red). Right, Photomicrograph of retrobeads (red) injection into SNr. ***C***, left, Diagram of stimulation with blue light on the LS to produce EPSCs on the MSN recorded by whole-cell patch clamp. Right, Location of pairs of cells recorded in the same slice into the striatum (from four animals). Each color depicts one pair of recordings. ***D***, Photomicrographs of a dMSN (left) and an iMSN (right). White lines depict the recording electrode. ***E***, The proportion of dMSN or iMSN cells that presented EPSCs in response to blue light stimulation of the M2→striatal-ChR2 axons. ***F***, Mean amplitude of EPSCs (pA) recorded on a dMSN (red) and iMSN (green). Inset, Representative EPSCs recorded in response to optogenetic stimulation of M2→striatal-ChR2 axons. The holding potential was –80 mV; the shaded region indicates SEM; *p = 0.02, Wilcoxon test. ***G***, Example of EPSCs recorded on a dMSN blocked by the AMPA antagonist CNQX. Download Figure 4-3, TIF file.

### Behavioral quantification during optogenetic inhibitions

The percentage of correct sequences of actions was quantified. A correct sequence was defined as the sequence with at least four presses on the first lever followed by at least four lever presses on the second lever. We also calculated the proportion of incorrect sequences (errors; Fig. 1*G*), by dividing the number of incorrect sequences by the total number of stimulation trials (on trials) or control light trials (off trials). Latency to initiate was calculated as the time between crossing the infrared beam out of the magazine and the first lever press in the sequences. The duration of a sequence was the time from the first press of subsequence 1 to the last press of subsequence 2. The transition between sequences was the time from the last press in subsequence 1 to the first press in subsequence 2. All animals were video recorded during the optogenetic manipulations. This allowed us to verify that all animals used both forepaws to execute the presses.

### *Ex vivo* whole-cell recordings

To express ChR2 in the M2 cortico-striatal projections, we injected 300 nanoliters of ChR2 into M2 (*n* = 4 mice). After 8–16 d, the animals were deeply anesthetized and transcardially perfused to obtain striatal slices as described in Díaz-Hernández et al. (2018). Postsynaptic currents in whole-cell configuration were evoked by pulses of blue light (1 ms), with a pair of cells (one dMSN and one iMSN) recorded per slice. The recordings were acquired as described in Díaz-Hernández et al. (2018).

### Experimental design and statistical analysis

Significance was determined by *p* < 0.05. For the proportions, paired or between groups, the χ^2^ test, the Wilcoxon test, or the Mann–Whitney *U* test was used, as appropriate. For comparisons along sessions, a non-parametric Friedman statistical test was used. When multiple comparisons were employed, a Benjamini–Hochberg correction was used to adjust the *p* value (Fig. 3*B*; Extended Data Figs. 3-1*A*, 3-2*G*). All statistical analyses were performed using GraphPad, R [R core Team (2013)] and MATLAB. Additionally, for the optogenetic manipulations experiments, we use estimation statistics based on confidence intervals (CIs) as described in Ho et al. (2019) and Manouze et al. (2019). The effect sizes and CIs are reported as: effect size [CI width lower bound; upper bound]. Five thousand bootstrap samples were taken, and the *p* value(s) reported are the likelihood(s) of observing the effect size(s), if the null hypothesis of zero difference is true. To account for multiple comparisons at each inhibition protocol, we considered a false discovery rate (FDR)-adjusted *p* value (*q* value) < 0.10, two-sided, as significant (Storey, 2002).

### Data and software availability

All data and MATLAB scripts will be available on request to the lead or corresponding authors.

## Results

To identify the premotor cortico-striatal contribution to serial order sequence execution, we developed a behavioral task in mice allowing us to perform two types of experiments. Mice were trained to execute two subsequences of lever presses on two levers in serial order (Fig. 1). For the first experiment, we aimed to identify whether neuronal activity in M2 or M1 is modulated during the task’s serial order sequences. We performed *in vivo* electrophysiological recordings in M2 or M1 to measure neuronal activity while animals executed these lever press sequences (Figs. 2, 3). For the second experiment, we aimed to identify whether the projections from these cortical regions contribute to the sequences’ execution, by optogenetically inhibiting the cortico-striatal projections either before or during the sequences’ execution, or during the transition between the two subsequences (Figs. 4-6).

### Mice can learn to execute serial order sequences of lever presses

To investigate the contribution of cortico-striatal synapses to the structuring and execution of self-paced serial order sequences, we trained mice in a serial order task to execute two subsequences of lever presses (Fig. 1*A*). The execution of a correct serial order sequence was achieved when animals performed at least four presses on a lever 1 (subsequence 1; S1) followed by four presses on lever 2 (subsequence 2; S2), which lead to the delivery of a reward in the magazine (pellet). To execute self-paced sequences, we first trained animals to execute forced sequences (forced: only one of the two levers was exposed at any point, signaling the animals when to press; Fig. 1*B*). Later in training, blocks of forced and self-paced serial order sequences were intercalated (self-paced: both levers remained exposed so that animals decided when to press and transition between subsequences; Fig. 1*C*). The requirement of intercalated blocks was necessary as animals’ performance dropped when they were required to perform only self-paced sequences (tested in a group of eight animals: data not shown). The main difference between a forced and a self-paced serial order sequence was that in the former, completing four presses on lever 1 retracted it and exposed lever 2; if four presses were executed on lever 2, it was also retracted, followed by the delivery of a pellet in the magazine (Movie 1). On the other hand, the execution of a correct self-paced sequence also required at least four presses on each lever, but in this case, both levers remained exposed throughout the self-paced trials (Movie 2).

Movie 1. Execution of a forced sequence. 10.1523/ENEURO.0173-21.2021.video.1

Movie 2. Execution of a self-paced sequence. 10.1523/ENEURO.0173-21.2021.video.2

A session containing both different kinds of sequences is identified in the figures as blocks of “forced–self-paced” sessions. During one of these sessions, the animals were required to achieve correct blocks of five forced and five self-paced sequences consecutively for 30 min. Figure 1*D* shows an example animal during the first (early) and the eleventh (late) day (rewards are labeled with filled red triangles for forced and in blue for self-paced). After 21 d of training, and 9 d performing blocks forced–self-paced sessions, animals showed a stable performance of correct sequences, demonstrated by the fact that performance no longer changed from sessions 9 to 11 [performance forced, χ^2^(2) = 2.51, *p* = 0.30, performance self-paced, χ^2^(2) = 1.0, *p* = 0.65, Friedman test, wild-type (WT) animals, *n* = 8, Fig. 1*E*]. No differences were found in comparisons of a number of task-relevant parameters between forced versus self-paced sequences: latency to initiate, the transitions intrasubsequences, the intervals between presses intra subsequences (*p* > 0.05, Mann–Whitney *U* test, WT animals, *n* = 8; Extended Data Fig. 1-1*A*).

While animals executing forced sequences reached an 83 ± 1.5% in correct performance (correct sequences/errors + correct sequences), the execution of self-paced sequences only reached 61 ± 1.7% (WT animals, *n* = 8; Fig. 1*E*, red and blue tick curves, respectively). To further investigate this performance, we quantified the types of errors and modes in which the animals executed correct sequences. An error was defined as a sequence of presses in which the animal did not achieve four presses on lever 1 followed by four presses on lever 2. The errors were grouped into four categories: (1) incorrect start (starting on the opposite lever); (2) breaks in S1; (3) premature switches from S1 to S2; and (4) breaks in S2 (Fig. 1*B*,*C*, right panels 1–4, respectively; note that 1 and three were only possible during self-paced). These four categories were quantified late in training (Fig. 1*G*). The decrease in Breaks in S1 and S2 was the primary determinant in performance improvement of both forced (ForcBreaks) and self-paced (S-PBreaks) sequences compared with pretraining (*p* < 0.05, Mann–Whitney *U* test, WT animals, *n* = 8; Extended Data Fig. 1-1*B*, panels 2, 4). A decrease in incorrect-start errors accounted for the major improvement in the self-paced performance (*p* = 0.007, Mann–Whitney *U* test, WT animals, *n* = 8; Extended Data Fig. 1-1*B*, panel 1). No difference in performance between forced and self-paced sequences was detected when accounting for only the available errors in both forced and self-paced sequences: BreaksS1+BreaksS2 (*p* = 0.5, Mann–Whitney *U* test, WT animals, *n* = 8; Fig. 1*E*, blue dashed curve vs red curve).

The correct execution in forced sequences was S1 = 4→S2 = 4; in self-paced the execution of correct sequences grouped in several options (as anything with S1 = >4→S2 >= 4 was correct). The majority of correct sequences were Long-S1 sequences (S1 > 4→S2 >= 4; WT animals, *n* = 8; Fig. 1*F*).

Besides the execution structure of the sequences, we quantified the length parameters. The latency to start, the transition between subsequences, the interpress intervals, the duration, all became faster as training progressed (Friedman test, *p* < 0.05; WT animals, *n* = 8; Extended Data Fig. 1-1*A*). Later in training, the latency to initiate the sequences was not different between forced and self-paced (forced: 1.1 ± 0.1 s, self-paced: 1.5 ± 0.3 s, *p* = 0.535, Mann–Whitney *U* test, WT animals, *n* = 8; Fig. 1*H*). Neither the transition time between subsequences (S1_end_→S2_start_) or the interpress intervals intersubsequences between the two modes (*p* > 0.05, Mann–Whitney *U* test; Fig. 1*J*,*K*). However, the duration and the number of presses were longer in the S1 of self-paced than in forced [forced_duration_: 3.2 ± 0.2 s vs self-paced_duration_: 4.8 ± 0.4 s, forced_S1presses_ = 4 ± 0 vs 6.1 ± 0.6 in self-paced_S1presses_, *p* = 0.0002, Mann–Whitney *U* test; Extended Data Fig. 1-1*C*,*D*).

This task parameterization showed that mice could learn and execute forced and self-paced serial order sequences in blocks. The main difference in the execution of these two modes of sequences (besides that self-paced have more possibilities for errors) is that animals execute longer chunks of presses in the subsequence 1 of self-paced sequences.

### Activity modulation in premotor and motor cortical neurons during the execution of serial order sequences

Once we established that mice are capable of executing serial order sequences, we questioned if, as predicted, the activity of M2 and M1 cortical neurons was modulated during the execution of these trained sequences (Tanji and Evarts, 1976; Mushiake et al., 1990; Churchland et al., 2006; Shima et al., 2007). For this purpose, we trained eight animals as in Figure 1. After 3 d in the blocks of forced–self-paced sessions, we performed brain surgeries to implant a mobile electrode array either in M2 (*n* = 6; Fig. 2*A*, middle panel) or M1 (*n* = 2; here, we verified the region to evoke forepaw movements by microstimulation; see Materials and Methods; Fig. 2*A*, right panel). After 4 d of recovery, the training restarted until animals reached a stable performance in the execution of forced and self-paced sequences (10–15 blocks sessions postsurgery). From these animals, 34 well-isolated units in M2 and 26 units in M1 were analyzed. Representative examples of these neurons’ activity during the executions of sequences are presented in Figure 2*B*,*C*. The *z* score heatmaps of Figure 2*D*,*E* show all recorded units during the execution of self-paced sequences (the same units during the execution of forced sequences are presented in Extended Data Fig. 2-1).

To answer whether the same units were active during the different phases of the sequences, we measured the mean activity of neurons above two *z* scores during the execution of the sequences. We classified neuronal activity into categories based on different parameters of sequence execution (Fig. 2*F*). We found no statistical difference in the comparison of M2 versus M1 activity, only a tendency for more modulated/engaged M2 neurons related to the initiation of a sequence (Fig. 2*G*). To explore this difference during sequence initiation, we compared the mean *z* score between M2 and M1 activity from the units that showed a significant modulation 1 s before starting the sequences (Fig. 2*H*,*I*). This comparison showed a bigger *z* score positive modulation in M2 than in M1 during the execution of the sequences (M2 = 14 of 34 units, M1 = 8 of 26 units; mean *z* score in self-paced_sequences_: M2_S1_ = 1.32, M2_S2_ = 0.85 vs M1_S1_ = 0.55, M1_S2_ = 0.22; *p* < 0.05, *z* score difference test; Fig. 2*J*, bars labeled “S1 and S2”). Remarkably M2 units showed a stronger modulation in self-paced sequences, even before the initiation of the sequence (M2_S-Pinit_ = 1.4 vs M1_S-Pinit_ = 0.8, *p* = 0.04 *z* score difference test; Fig. 2*J*, bars labeled “Init” in blue) and a similar tendency during the initiation of forced sequences (Extended Data Fig. 2-1*H*, bars labeled “Init” in red). No difference in the number of recruited units throughout the execution of the sequences was detected (*p* > 0.05, χ^2^ test; Extended Data Fig. 2-1*I*).

### Both M2 and M1 contain units encoding the length of the serial order sequences of actions

After the evaluation of the mean *z* score related to the execution of the serial order sequences, we tested the hypothesis that M2/M1 encode the length parameters of the execution of these sequences (Riehle and Requin, 1989; Riehle et al., 1994; Lucchetti and Bon, 2001; Sakurai et al., 2004; Churchland et al., 2006; Jin et al., 2009; Hernández et al., 2010; Merchant et al., 2013; Crowe et al., 2014; Murakami et al., 2014; Mendoza et al., 2018). To evaluate this possibility, we performed linear regressions between the duration of the segments of the sequences (total sequence, S1, transition, or S2) and the neural activity recorded from individual neurons in either M2 or M1. We reasoned that if these cortices contained activity encoding the sequence length, their cortico-striatal projections could convey this information to the striatum.

Figure 3*A* shows two examples of units that presented significant regressions (*R*^2^ > 0.6 and *p* < 0.05) in specific time bins during the execution of serial order self-paced sequences (Fig. 3*A*, middle row panels, gray shadows). Figure 3*B* shows the proportion of units that presented with significant regressions based on five questions: column i, does the activity before the first press (1 s) encode the sequences’ length? ii, does the mean activity during the sequence encode the length of the sequence? iii, iv, and v, Is there a relationship between the duration of S1, the transition or the duration of S2, and the mean firing rate during these epochs? The answer to these five questions is presented per recorded unit in either M2 or M1 (Fig. 3*B*, upper panels) and as the % of units with significant regressions per region (Fig. 3*B*, bottom panels). Notably, the biggest proportion of units with significative regressions was between the sequence duration and the firing rate before the initiation of the sequence in both M2 and M1 (Fig. 3*B*, columns i) or the sequences duration and the mean firing rate of M2 during the sequences (Fig. 3*B*, columns ii).

The percentage of recruited units over time (units presenting significant regressions, 200-ms bin, sliding 10 ms; *R*^2^ > 0.6 and *p* < 0.05) is presented in Extended Data Figure 3-1*B*. Despite small tendencies, no difference was detected in the proportion of units recruited overtime either when comparing M2 versus M1 nor when comparing within each region between forced and self-paced sequences (1 s before or one after the start of the sequences). Similarly, no differences were found between neuronal activity and the duration of the sequence, duration of S1, duration of the transition, or the number of presses (comparison of bars *p* > 0.05, χ^2^ test; Extended Data Fig. 3-1*B*).

To address whether specific cortico-striatal M2 or M1 neurons encode for sequence parameters, we recorded from antidromic photo-identified cortico-striatal neurons *in vivo* (Lima et al., 2009; Díaz-Hernández et al., 2018). To achieve this, we first confirmed the lateral region of the striatum (LS) that is innervated by both M2 and M1 projections (Extended Data Fig. 3-2*A–D*; Oh et al., 2014; Sohur et al., 2014; Hintiryan et al., 2016; Hunnicutt et al., 2016). Then we trained mice in which the expression of Channelrhodopsin-2 (ChR2) was targeted to the excitatory cortical neurons in M2 (*n* = 4) or M1 (*n* = 2) and their projections to the striatum (using Emx1-Cre mice; Guo et al., 2000; Gorski et al., 2002). We then implanted an electrode array above the ChR2 expression and an optical fiber into LS to antidromically activate the cortico-striatal units (Extended Data Fig. 3-2*E–G*; Friedman et al., 2015). Following this procedure and after a stable performance in the execution of blocks of forced–self-paced sequences, the activity of M2 or M1 units was recorded. During these recordings, we recorded cortical and cortico-striatal photo-identified units (PID units). A PID unit was that, at the end of the session, responded to antidromic light stimulation with short latency (<10 ms; Díaz-Hernández et al., 2018). The mean latency of M2+M1→LS was 5.1 ± 0.4 ms; *n* = 21; Extended Data Fig. 3-2*F*, bottom right panel). Following these criteria, we identified 10 M2 and 11 M1 cortico-striatal PID units (Extended Data Fig. 3-2*G*). As in the non-PID units (Fig. 3*B*), the major proportion of PID units presented significant regressions between the sequences duration and the firing rate before the initiation, the duration, and the mean firing rate during the execution of the sequences (Extended Data Fig. 3-2*G*, columns i, ii).

In summary, in Figures 2, 3 and Extended Data Figure 3-2, we show that the activity of M2 and M1 is related to the execution of the trained sequences. The mean *z* score analysis showed that M2 had a bigger modulation than M1 during the execution and even before the initiation of the serial order sequences. Furthermore, the regression analysis showed that both M2 and M1 contain units that encode the sequences’ execution length.

### Inhibition of lateral striatal neurons before initiation of a serial order sequences impairs its execution

This study’s main goal was to establish whether cortico-striatal projections of the premotor and motor cortex contribute to the execution of sequences. To address this point, we first verified that neuronal activity in the lateral striatal (LS) region that receives inputs from M2 and M1 (Extended Data Fig. 3-2*A–D*) contributes to the execution of serial order sequences. For this purpose, we trained a group of animals in which the inhibitory opsin archaerhodopsin (Arch3.0) was injected bilaterally into LS followed by fiber-optic implantation above the injection sites (Extended Data Fig. 4-1*A*). Then, once the animals reached around 80% success (correct sequences) in the performance of forced and 60% in self-paced sequences (Extended Data Fig. 4-1*A*, panel 3), we performed sessions of optogenetic inhibition, delivering the light to inhibit Arch3.0-expressing neurons in a state-dependent manner during the execution of the sequences. We randomized three protocols (each protocol on a different day): (1) before the initiation; (2) during the execution; or (3) during the transition between subsequences (Extended Data Fig. 4-1*B–G*, *H–M*, *N–S*, respectively). We observed that optogenetic inhibition before starting the sequence delayed the initiation (Extended Data Fig. 4-1*C*). Inhibition during the execution of the sequence increased premature switches (Extended Data Fig. 4-1*J*, panel 3) and decreased the correct Long-S1 self-paced sequences (Extended Data Fig. 4-1*K*). Inhibition during the intersubsequence transitions increased the transition time between forced subsequences and showed a tendency to increase the time between self-paced subsequences (Extended Data Fig. 4-1*R*). Note that the data for the forced sequences are only presented in the figures when significant effects were detected; otherwise, they are presented only in Table 1.

### Presequence initiation inhibition of either motor or premotor cortico-striatal projections impairs initiation, but only the latter disrupts the execution

Once we verified that direct inhibition of LS neurons impaired the execution of sequences, we asked whether the premotor (M2→LS) or the motor (M1→LS) cortico-striatal projections contributed to the execution of these sequences. We first verified the inhibition of striatal neurons by the optogenetic inhibition of cortico-striatal projections using Arch3.0 with 2-s pulses of light (Fig. 4*A*). This pulse length is in a proper range far from the biophysical constraints of using Arch for optogenetic inhibition (Mahn et al., 2016; Díaz-Hernández et al., 2018). Furthermore, we evaluated the proportion of labeled cells in M2/M1 that contain corticofugal axons crossing by the area of the LS manipulations (Extended Data Fig. 4-2).

Next, we tested whether the contribution of cortico-striatal projections is time dependent (Bartolo et al., 2014; Bartolo and Merchant, 2015; Li et al., 2016; Nakayama et al., 2018). We performed optogenetic inhibitions of the M2→LS (*n* = 9) or M1→LS (*n* = 8) projections in a state-dependent manner, using three protocols: (1) before initiation (Fig. 4); (2) during execution (Fig. 5); and (3) during the transition between subsequences (Fig. 6).

Most of the effects of optogenetic inhibition were on self-paced sequences. Therefore, for Figures 4-6, forced sequences are only presented when effects were detected; otherwise, only self-paced sequences are presented. The full data for forced and self-paced inhibitions is in the Table 1.

To carry out these experiments, a group of Emx1-Cre mice was subject to bilateral Arch3.0-eYFP expression into M2 or M1 (or eYFP for control animals) and bilateral fiber optic implantation into the LS (Fig. 4*B*; see Figs. 4-6). After surgery, animals were allowed to recover for 3 d before training started and continued until the performance was stable (Fig. 4*D*). During the optogenetic inhibition sessions, and depending on the protocol, a continuous pulse of green light was applied randomly in 50% of the trials (2 s; triggered by the behavior), allowing us to compare the effects of light inhibition on each animal within the same session.

For the inhibition “before” initiation protocol, we aimed to assess whether the cortico-striatal projections contribute to sequence execution by interfering with the initiation/preparation of the sequences for which we took advantage of the stereotyped behavior of the trained animals. An infrared beam was placed between the magazine and the levers (red arrow-dashed line coming out of the magazine; Fig. 4*C*), it was possible to trigger a pulse of light when the animal crossed the infrared beam before starting the sequences (Tecuapetla et al., 2016).

The inhibition of the M2→LS projections before initiation increased premature switches (M2→LS_Self-paced_on = 20 ± 5 vs M2→LS_Self-paced_off = 8 ± 1; the paired mean difference was 12.2 [95.0%CI 6.8, 27] and *p* = 0.0001, *q *=* *0.01 two-sided permutation *t* test; Fig. 4*E*, panel 3, light blue data; Movie 3). This effect was not observed in control animals (Δ on-off comparison to the control group: the unpaired mean difference between control and M2→LS was 10.4 [95.0%CI 3.7, 2.4]. The *p* value of the two-sided permutation *t* test was 0.0224, *q *=* *0.06; Fig. 4*F*, panel 3, bottom part). The increased premature switches were accompanied by decreased presses within the self-paced sequence (M2→LS_Self-paced_on = 9 ± 0.6 vs M2→LS_Self-paced_off = 10 ± 0.4; the paired mean difference was −1.16 [95.0%CI −2.42, −0.495], *p* = 0.016, *q *=* *0.05 two-sided permutation *t* test; Fig. 4*F*, light blue data).

Movie 3. Example of a broken sequence after light inhibition before the initiation of the sequence. 10.1523/ENEURO.0173-21.2021.video.3

This inhibition protocol also increased the latency to start the sequences (M2→LS_Self-paced_Latency_on = 2.9 ± 0.4 vs M2→LS_Self-paced_Latency_off = 1.8 ± 0.3; the paired mean difference was 1.1 [95.0%CI 0.556, 1.96], *p* = 0.0026, *q *=* *0.01 two-sided permutation *t* test; Fig. 4*G*, light blue left panel; forced sequences were also delayed, see Table 1, data related to Fig. 4), an effect not observed in control animals (Fig. 4*G*, Δ on-off panel, gray data). This increased latency to start was accompanied by an increase in the animals’ return to the magazine in the trials that received light inhibition, suggesting that inhibition interrupted the proper serial order sequence initiation (see Movie 4; M2→LS_SP-returns_to_start_on = 9 ± 2% vs M2→LS_SP-returns_to_start_off = 1 ± 0.7%, the paired mean difference between control and M2→LS was 8.29 [95.0%CI 5.14, 11.7], *p* = 0.006, *q *=* *0.03 two-sided permutation *t* test. Unpaired mean difference between control and M2→LS was 9.86 [95.0%CI 6.0, 13.8], *p* = 0.0002, *q *=* *0.002 two-sided permutation *t* test; Fig. 4*G*, Δ on-off panel).

Movie 4. Example of return to start after light inhibition before the initiation of the sequence. 10.1523/ENEURO.0173-21.2021.video.4

Conversely to the inhibition of M2→LS projections, the increase in premature switches and the decrease in the number of presses within the sequence were not observed when we inhibited M1→LS projections (Fig. 4*E*,*F*, dark blue data). Instead the inhibition of the M1→LS projections increased the latency to start the self-paced sequences (M1→LS_Self-paced_Latency_on = 3.2 ± 0.4 vs M1→LS_Self-paced_Latency_off = 2.6 ± 0.3; the paired mean difference was 0.614 [95.0%CI 0.40, 1.1] and *p* = 0.009, *q *=* *0.04 of the two-sided permutation *t* test; Fig. 4*G*, comparison to control: panel Δ on-off same figure, dark blue vs gray data, unpaired mean difference between control and M1→LS was 0.88 [95.0%CI 0.4, 1.7] and *p* = 0.003, *q *=* *0.01 of the two-sided permutation *t* test), and the returns to start (comparison to control: panel Δ on-off in the same figure, dark blue vs gray data; unpaired mean difference between control and M1→LS was 11.8 [95.0%CI 3.3, 26.1] and *p* = 0.02, *q *=* *0.06 of the two-sided permutation *t* test; Fig. 4*H*).

Together these results suggest that the M2→LS but not the M1→LS projections contribute to the execution/structuring of self-paced sequences while both M2→LS and M1→LS contribute to the initiation.

### Inhibition of premotor cortico-striatal projections at the start of the execution decreased Long-S1 sequences

One prediction for the cortico-striatal projections’ contribution to serial order sequences execution is that their requirement would be time dependent (see Figs. 2, 3; Li et al., 2016; Nakayama et al., 2018). To further prove this idea, we performed a second protocol: inhibition during “execution,” which consisted of performing light inhibition of the cortico-striatal projections once the execution of the sequences started (triggered by the first press in S1; Fig. 5*A*). Using this protocol, we observed that the inhibition of the M2→LS projections did not modify the proportion of the different categories of errors (Fig. 5*B*) nor the number of presses in the sequences (Fig. 5*C*), but it did decrease the proportion of Long-S1 sequences (M2→LS_Long_S1_Seq_on = 35 ± 5 vs M2→LS_Long_S1_Seq_off = 50 ± 6, the paired mean difference was −14.6 [95.0%CI −18.9, −9.8] and *p* = 0.002, *q *=* *0.04 of the two-sided permutation *t* test; Fig. 5*D*, upper panels; comparison to control eYFP-group: Δ on-off panel, unpaired mean difference between control and M2→LS was −18.4 [95.0%CI −28.3, −8.8] *p* = 0.003, *q *=* *0.04 of the two-sided permutation *t* test Fig. 5*D*, bottom panel), with no overall effect on the duration or the number of presses in the sequences (see Table 1; data related to Fig. 5). The inhibition of the M1→LS projections did not yield any significant effects with the inhibition during execution protocol (Fig. 5, dark blue data).

The results from the inhibition of M2→LS or M1→LS projections during the beginning of the sequence execution highlight that the activity of the M2→LS projections is important for the length of the sequences (particularly for S1). Furthermore, these results also suggest that, at least later in training, the M1→LS projections are not required for sequence execution.

### Inhibition of premotor cortico-striatal projections during the transition between serial order subsequences increases the transition time

So far, we have shown that the cortico-striatal projections from M2→LS contribute to the appropriate initiation and execution of self-paced serial order sequences (Figs. 4, 5). However, in the previous experiments, the light inhibition never occurred during the transition between subsequences. Given that a small proportion of units from either M2 or M1 showed significant modulation during the transition-moment (Fig. 2*F*,*G*), we asked whether inhibiting directly during the transition could reveal whether the M2→LS or M1→LS contribute to the transition. For this purpose, we set up an inhibition during “transition” protocol, in which the press before the penultimate press of S1 triggered light inhibition for 2 s (Fig. 6*A*, for self-paced; in forced sequences, it was the third press; see Table 1, data related to Fig. 6).

Figure 6*B* shows the mean press in which the animals of the M2→LS or the M1→LS groups received the inhibitory stimulation (or the corresponding timestamp in the “off” trials); on average, it was the press −2 counting from the final press in the S1 (M2→LS_transition light_on = −2.0 ± 0.1 vs M2→LS_transition light_off = −1.9 ± 0.1, *p* = 0.25, Mann–Whitney *U* test; Fig. 6*B*). With this protocol, we observed that the inhibition of M2→LS, but not M1→LS, showed a tendency to increase the duration of the transition (M2→LS_Transition_on = 1.0 ± 0.06 vs M2→LS_Transition_ off = 0.8 ± 0.06, the paired mean difference was 0.08 [95.0%CI 0.02, 0.16] and *p* = 0.050, *q *=* *0.4 of the two-sided permutation *t* test; Fig. 6*C*, upper panels), that reached significance when compared with the eYFP-control group: unpaired mean difference between control and M2→LS was 0.18 [95.0%CI −0.09, 0.3] and *p* = 0.003, *q *=* *0.03 of the two-sided permutation *t* test; Fig. 6*C*, Δ on-off bottom panel). This effect was time-specific since neither the inhibition before nor the inhibition execution protocol on the M2→LS yielded a similar effect (Fig. 6*D*). This effect on the transition raised the question of whether the M2→LS projections treat each subsequence as independent chunks or concatenate them into a single chunk once the whole sequence has been acquired (Geddes et al., 2018; Martiros et al., 2018). We hypothesized that if the transition was affected by the light inhibition, the proportion of breaks in the sequences might increase as well. Contrary to this hypothesis, no modifications on the breakings of sequences were detected by this protocol (Fig. 6*E*). We also observed no differences in the number of presses or sequence length (Table 1), supporting the idea that the M2→LS projections contribute to the transition intersubsequences without affecting the second part of the serial order sequence.

Altogether, the results from the recording and inhibition experiments reveal that the cortico-striatal projections to the LS from the premotor and motor cortices have specific contributions to the execution of sequences. Both projections contribute to the initiation, while M2→LS also contributes to the correct execution and transition between subsequences of self-paced sequences. Importantly the M2→LS contribution was essential during the beginning of the execution, suggesting its contribution is relevant before the initiation, at the beginning of execution, and decreasing in relevance as the sequence execution progressed.

### Premotor cortico-striatal projections innervate the direct pathway with a stronger synaptic weight than the indirect pathway

Finally, we investigated whether the M2→LS projections differentially impact the two subcircuits of striatal projection neurons, the direct or indirect striatal pathways (Wall et al., 2013), by looking at synaptic connectivity. For these experiments, we crossbred Emx1-Cre (allowing us to express ChR2 in M2) with BAC D2-GFP mice. By injecting red retrobeads into the substantia nigra, we could identify in the striatum neurons from direct pathway (red retrobeads; dMSN), or the indirect pathway (GFP; iMSN). This allowed us to record from identified striatal neurons in brain slices *ex vivo* while stimulating the M2→LS ChR2 axons (Extended Data Fig. 4-3). Using this procedure, we recorded pairs of neurons in the same brain slice in the LS (one iMSN and one dMSN; counterbalancing the recording order per slice). We evoked postsynaptic responses by activating the M2→LS ChR2 projections with brief pulses of light (1 ms; Extended Data Fig. 4-3*C*). From these experiments, we recorded a total of 6 pairs of striatal neurons (in 6 different slices from four animals). Postsynaptic evoked responses had a bigger amplitude in the dMSN than in the iMSN (dMSN: 290 ± 100 vs iMSN = 30 ± 9 pA, Mann–Whitney *U* test, *p* = 0.031; Extended Data Fig. 4-3*F*), with no difference in their latencies (dMSN: 3.3 ± 0.1 ms vs iMSN = 3.8 ± 0.1 ms, Mann–Whitney *U* test, *p* > 0.05; Extended Data Fig. 4-3*F*). These evoked postsynaptic responses were glutamatergic, being blocked by the AMPA receptor antagonist CNQX (Extended Data Fig. 4-3*G*). This finding suggests that the M2→LS projections may have stronger synaptic connections onto the dMSNs than onto the iMSNs *in vivo* when the animals are executing the serial order action sequences.

## Discussion

These results show that mice can execute serial order forced and self-paced sequences of lever presses (Fig. 1). Both the premotor (M2) and motor (M1) cortices are modulated during sequence execution (Fig. 2). M2 showed a bigger modulation during the initiation and execution in the units modulated before the start of the sequences (Fig. 2*J*). Both M2 and M1 units showed regressions between their activity and the sequence duration before or during the execution (Fig. 3*B*), even in photo-identified M2 and M1 cortico-striatal cells (Extended Data Fig. 3-2*G*). The M2→LS and M1→LS projections contribute to the proper initiation, but mainly M2→LS projections contribute to the structuring/execution of serial order self-paced sequences (Figs. 4-6). The M2→LS synapses present bigger amplitude postsynaptic responses onto the direct versus the indirect pathway of striatal neurons (Extended Data Fig. 4-3).

Mice had been previously been shown to develop and execute serial order operant tasks, including pressing two levers (Geddes et al., 2018; Rothwell et al., 2015). The task adapted here allows temporally separated manipulations within the different phases of the two subsequences (initiation, execution, and transition between subsequences). We first trained animals in forced sequences and later in blocks of forced–self-paced sequences as animals trained only in the latter dropped their behavior. The fact that animals were more efficient at executing forced than self-paced can be explained by the greater number of possible errors in self-paced sequences. However, a difference in the recruitment of brain structures in forced versus self-paced sequences is not excluded (Romo and Schultz, 1992; Hernández et al., 2010).

To identify whether the cortico-striatal projections from M2/M1 contribute to sequence execution, we investigated whether neuronal activity in these structures was modulated during the execution of the sequences. We identified that M2 and M1 units showed activity modulations milliseconds before the start and during the execution of these serial order sequences (Figs. 2, 3), with mostly exclusive firing categories (Fig. 2*F*). Importantly, the units from M2 that were modulated before the sequences started showed a bigger positive modulation during the execution and before starting the sequences than those from M1 (Fig. 2*J*). This increased activity time before the execution of movements is attributed to time estimation or preparation to execute an action [e.g., the anticipatory activity (ramping) in M2 has a direct relationship with the time that animals wait to start an action or the duration of a stimulus (Murakami et al., 2014; Mendoza et al., 2018)]. In this study, the animals had to approach the lever before the first press in the sequence. We could not therefore rule out that the activity before starting the sequences may be related to motor preparation, although the inhibition of this activity before pressing delayed the latency of both forced and self-paced sequences, but only impaired the execution of self-paced sequences. Together, these results show that units in M2/M1 are modulated during the execution of sequences. When evaluating whether these units’ activity may encode the execution parameters, we observed no significant differences in the proportions of units in M2 versus M1, as measured by regression analysis between neuronal activity and temporal parameters of the sequences (Fig. 3*B*). These results show that both M2 and M1 contain units encoding the sequences’ execution (Nelson et al., 2020; Berlot et al., 2021). However, it must be acknowledged that the lack of differences in some of the comparisons between M2 and M1 may be influenced by the small set of recorded cells (34 cells from M2 and 26 from M1).

To evaluate how the M2/M1 cortico-striatal projections contribute to the execution of serial order sequences, we performed two experiments. First, we recorded from cortico-striatal antidromic PID units (Extended Data Fig. 3-2). Second, we performed state-dependent temporal inhibitions of the M2→LS or the M1→LS projections either before or during the execution of sequences (Figs. 4-6). Although the photo-identification of cortico-striatal M2→LS or M1→LS was low (10 per structure), it allowed us to detect units in each of these cortices that presented significant regressions with temporal parameters of the execution of the sequences. However, there were too few photo-identified neurons to infer whether M2 or M1 has a more important contribution to sequences execution. The contribution of these cortico-striatal projections was thus mainly evaluated with state-dependent optogenetic inhibition.

To test the hypothesis that the M2/M1 cortico-striatal projections have time-specific contributions to sequences execution, we performed three protocols of inhibition while animals performed lever pressing sequences. Our inhibitions were all 2 s, below the safe limit of the biophysical constraints of using Arch for optogenetic inhibitions (Mahn et al., 2016).

Interestingly, the inhibition of either the M2→LS or M1→LS, before the initiation increased the latency to start the sequences (Fig. 4*G*). This increase in latency was accompanied by an increase in the animals’ return to the magazine in the initiations that received light inhibition, as if the inhibition interrupted the proper initiation (Fig. 4*H*; see Movie 4). These results are consistent with a model where the M2→LS and M1→LS cortico-striatal projections are required for the proper initiation of sequences, with the former likely setting up the parameters for the upcoming sequence (for further discussion, see below).

Remarkably, the three inhibition protocols revealed that the projections from M2→LS, but not the M1→LS, are temporally required for the execution of self-paced sequences. The inhibition of M2→LS before the start altered the sequence structure, increasing premature switches (Fig. 4*E*) and decreasing the number of actions in the sequence (Fig. 4*F*). Their inhibition during the beginning of the execution affected the first segment of the sequence (by decreasing the probability of executing Long-S1 sequences; Fig. 5*D*). Their inhibition during the transition increased the transition time between subsequences, although this last had the slightest effect (Fig. 6*C*).

A previous experiment, which decreased the M2→striatal projections (by manipulating M2→striatal projections plus their collaterals to other brain regions) during the execution of a serial order short sequence reported that inhibition of M2→dorsolateral striatal cells, retrogradely labeled from the striatum, impaired the first step accuracy (Rothwell et al., 2015), perhaps by increasing incorrect starts or promoting premature switches from S1 to S2. Here, the possibility of longer subsequences in the sequence allowed for measuring incorrect starts, breaks, premature switches, and the transition time between subsequences. Our inhibitions of M2→LS was spatially and temporally specific (if the optogenetic inhibition does not backpropagate as it has been documented; El-Gaby et al., 2016), showed no effect on incorrect starts. Rather we observed an increase in premature switches when inhibiting before the start, a decrease in Long-S1 sequences when inhibiting during the execution, and slowness of the transition time when inhibiting around the transition.

Another important point reported here is the specificity of the effects mainly on self-paced sequences (see Table 1). How is it that the premotor projections can differentiate between the execution of forced and self-paced sequences? A possible explanation is that the M2→LS projections could detect whether the animals were in blocks of self-paced sequences, engaging these projections with a bigger sensitivity to the inhibitions as a consequence of self-deciding the execution. This possibility is supported by previous studies showing that when animals engage in self-paced behaviors, as opposed to forced behaviors, some cortical subcircuits become engaged and more important (Romo and Schultz, 1992; Hernández et al., 2010; although see Kurata and Tanji, 1985; Thaler et al., 1988). An idea supported by the tendency of cortico-striatal M2 PID units to show a bigger proportion of units with significant regression in the self-paced sequences (Extended Data Fig. 3-2*G*, columns i, iii). Importantly we observed that the inhibition of the M1→LS did not affect the execution of sequences, consistent with the idea that M1→LS activity is not required to execute sequences once these have been learned (Kawai et al., 2015).

Finally, another remarkable finding from the optogenetic inhibitions experiments is that the M2→LS projections may contribute as a serial driver of sequence execution (Lashley, 1951; Zeigler and Gallistel, 1981; Graybiel, 1998; Rosenbaum et al., 2007; Jin and Costa, 2015). Supporting this idea, their inhibition before the starting could restart the initiation, increasing premature switches during the execution, and decreasing the number of actions (Fig. 4*E*,*F*). Conversely, their inhibition during the start of the execution decreased the probability of executing Long-S1 sequences only (Fig. 5*D*).

In addition to the inhibition of cortico-striatal projections, we verified the LS requirement for the sequence execution and addressed the possibility that our cortico-striatal inhibitions may affect other brain areas besides the LS by performing two experiments: (1) we directly inhibited the lateral striatal neurons with the same protocols that inhibited the cortico-striatal projections (Extended Data Fig. 4-1); and (2) we evaluated whether the manipulated region contains axons en passant from M2/M1 that reach other brain areas (e.g., thalamus/pons; Th/Pns; Extended Data Fig. 4-2).

The first experiment’s results found that direct inhibition of LS during sequence execution decreased the length of correct sequences (Long-S1 sequence) and the number of presses (Extended Data Fig. 4-1*K*,*M*). The inhibition of the LS in the transition protocol increased the transition time between subsequences (Extended Data Fig. 4-1*R*). These effects were in line with the inhibition of M2→LS projections. However, the LS inhibition before the sequence start did not change the errors in self-paced sequences, contrary to the inhibition of the M2→LS projections which increased premature switches with this protocol. A possible explanation for this last difference is that M2→LS activity may be required to set the execution of sequences by impinging on specific striatal subcircuits (Wall et al., 2013), However, our direct inhibition of the LS did not differentiate between striatal subcircuits. To address this, Extended Data Figure 4-3 shows that the M2→LS innervates, strongly the striatal projection neurons from the direct pathway. Such preferential innervation has been previously suggested (Wall et al., 2013; Nelson et al., 2020) to direct the structuring of sequences in serial order tasks (Rothwell et al., 2015), although only after learning.

Lastly, the quantification of the cells that project from M2/M1 to other brain areas by crossing through the LS showed it to be a small percentage of neurons (M2/M1→Str→Th 3%; M2/M1→Str→Pns 3%; Extended Data Fig. 4-2). This data, plus the evidence that Arch3.0 mainly has inhibitory actions through local inhibition rather than affecting the propagation of action potentials (El-Gaby et al., 2016), suggests that the effects observed here were predominantly from optogenetic inhibition of the cortico-striatal projections from M2 or M1 into the LS.

In conclusion, this study shows that the premotor cortico-striatal projections to the LS contribute to the initiation and execution of self-paced sequences. It supports a model in which both M2 and M1 contain activity modulated by sequence initiation and execution. However, the M2→LS cortico-striatal projections mainly contribute to the proper execution/structuring of self-paced sequences. Both, M2 and M1 cortico-striatal projections contribute to the initiation of sequences. Also, our findings support the idea that the premotor cortico-striatal projections to the LS are a serial driver for the execution of sequences (Lashley, 1951; Zeigler and Gallistel, 1981; Graybiel, 1998; Rosenbaum et al., 2007; Jin and Costa, 2015). Altogether the presented findings may have important implications for pathophysiological conditions whereby self-paced generation of actions is impaired.

## Synthesis

Reviewing Editor: Karen Szumlinski, University of California at Santa Barbara Decisions are customarily a result of the Reviewing Editor and the peer reviewers coming together and discussing their recommendations until a consensus is reached. When revisions are invited, a fact-based synthesis statement explaining their decision and outlining what is needed to prepare a revision will be listed below. The following reviewer(s) agreed to reveal their identity: NONE.

In consultation with two experts reviewers, we have reached the decision of “Revise and Re-review” as the suggested revisions to your report should require less than two months' work. Overall, we agree that the results of this report are very interesting, with novel findings on the control of lever-pressing behavior by cortico-striatal projections coming from M2. We all were impressed by the immense amount of data presented in this report and commend the authors for their efforts. However, the report, as currently written, is very dense and reviewers raised some organizational issues. Thus, the major suggestion put forth by both reviewers relate to slimming down the amount of data in the main text by placing the extraneous data into a supplement to facilitate interpretation of the key findings. Reviewer 1 raised the suggestion of possibly dividing the report into 2 reports; however, upon discussion, we agreed that the report should remain as one, and suggestions are provided by the reviewers regarding what information should be moved to the supplement. While the suggestions by the reviewers should take less than 2 months to complete, these are still challenging times and eNeuro understands if more time is required. If you do find yourselves needing more time to address the reviewers' concerns, please do not hesitate to reach out to the journal to ask for an extension. Please find below the specific comments of each reviewer.

Reviewer 1:

This is an interesting, well executed and analyzed set of data examining whether there is 1)sequence representation in primary M1 and secondary motor cortex M2 in mice. While prior works have suggested sequence encoding in these structures, there has also been recents works showing that these structures were not functionally necessary. To shed more light on this discrepancy, here the authors did a very thorough job of investigating the minutia of sequence behavior they are observing, recording M1 and M2 activity during both forced and choice sequences, optotagging recorded neurons to show that they project to striatum, and doing careful optogenetic inhibition of M2 or M1 inputs into striatum to look at functional contribution. At each step, the technique is validated. The finding that M2 may support the initiation of sequence, with its contribution reducing during execution supports a hypothesis of limited involvement, with less contribution by M1 output to striatum.

The main complaint of this paper is that it is very dense. My recommendation would be to split it into two separate companion papers for publication in eneuro; the first the recording findings followed by 2) the optogenetic inhibition studies. There is a large amount of data here, and even with this suggestion there could be some pairing down. The authors present us with everything, and understandably they want to tell the complete picture.

However, I do wonder whether there are places where combining data would make it easier for the reader without changing the interpretation. In regards to this part, I am not sure what the final experiment (Figure 7) has to say about the beginning which is investigating M1 and M2 inputs. This could be removed, but understand if the authors are hesitant to do so. There are also places where taking absolute values (absolute of positive and negative beta coefficients)may make it easier on the reader without loosing the interpretation. The averaged z-score of all neurons combined need not be presented.

Overall- this paper reads fairly well- there is just an insane amount of data to keep track of. Hypotheses are stated, statistics appear to be corrected for number of comparisons when necessary. While they have a limited number of neurons they recorded from, this was in part due to the optogenetic tagging and care they took in the recordings, which is appreciated.

Reviewer 2

This study investigates the role of corticostriatal connections in the control of the initiation and execution of movement sequences in the mice. The animals were trained to execute two subsequences of lever presses that were forced or self-paced. Neural recordings in M1 and M2 as well as state-dependent optogenetic inhibitions of their cortico-striatal projections were carried out during task performance. The results showed that inhibition of cortico-striatal projections, specially from M2, produces changes in the initiation of self-paced sequences.

This is an interesting study providing novel results on the contribution of cortico-striatal projections coming from M2 on the control of sequences of self-paced lever presses using optogenetic manipulations. Nevertheless, I have a set of main comments that needs to be addressed before publication. First, the paper is very convoluted, and it is difficult to extract the take home message from detailed information that is not contributing to the main story. I suggest that all the neurophysiological and optogenetic results on forced sequences could be moved to supplementary information. This will make the figures less busy and the flow of ideas cleaner. I also suggest that panels E and the right portion of panels H, J and K of Figure 1 should be reported in the supplementary information. Second, the number of recorded cells is small (34 M2, 26 M1), almost bordering on an unacceptably small database to determine neurophysiological properties of an area during task performance. A clear precaution note about the number of recorded cells is needed in the discussion. In addition, the analysis on the bottom panels of Figure 2F and G is performed on a small population of heterogenous single cells responses and, hence, I am not sure is valid to compare the z-scores of the two areas. I also suggest of send Figures 2L and M to the supplementary material. In contrast, panels J and K of Figure 2 are important. It is not clear whether the regression analysis of Figure 3 was performed using a permutation test across trails and sliding windows. Furthermore, the duration and the # of presses in a sequence covary, so it is not evident why a neuron should be tuned to the duration or the number of executed movements. I suggest moving the bottom part of Figure 3B and Figure 3C to the supplementary information. Third, the present study tests optogenetic manipulation effects in different tasks epochs on a large set of behavioral parameters. A multivariate false positive rate should be considered for the description of the results. Additionally, the effects on Figure 4E.3 and 4F.1 seem to be due to an outlier animal, please check the effects after outlier removal. Finally, it is difficult to see the relevance of the synaptic connectivity ex vivo experiments. The link between the stronger synaptic connections from M2 to LS into dMSN and the sequential behavior is indirect.

Minor comments.

The paper has many grammatical and syntactic errors and should be proofread by a native speaking English editor.

Please rewrite this long sentence: We show that while both M2 and M1 contain activity modulations related to the execution of serial order self-paced sequences, the predominant cortico-striatal contribution to the proper execution/structuring of self-paced serial order sequences comes from the premotor cortex.

I am not sure this is adding too much to the story: In M1, we trained more animals but show recording data only from the two verified to evoke forepaws movements by M1 microsimulation (see methods).

The effect of optogenetic inhibition in LS before starting the sequence on the probability of breaking subsequence 2 on Self-paced trials seem to be an outlier effect.

How many animals were used for the experiments of figure 4 to 6.

It is crucial to refer to the studies of Jun Tanji and RE Passingham on the control of self-initiated versus externally triggered movement and sequences. Also, referring to the neurophysiology of SMA/preSMA (Merchant et al., 2013, Crowe et al., 2014) and the putamen (Bartolo et al., 2014; Bartolo and Merchant , 2015) during the rhythmic and arhythmic execution of tapping sequences is in place.

Typos.

microsimulation

line 622 for figures 4-6,

line 740 Importantly the M2 LS contribution was essentially
