# Supplementary figures and images for: The Contribution of Premotor Cortico-Striatal Projections to the Execution of Serial Order Sequences
Source: eNeuro. 2021 Sep 21;8(5):ENEURO.0173-21.2021. doi: 10.1523/ENEURO.0173-21.2021 (PMC8457420; doi:10.1523/ENEURO.0173-21.2021)

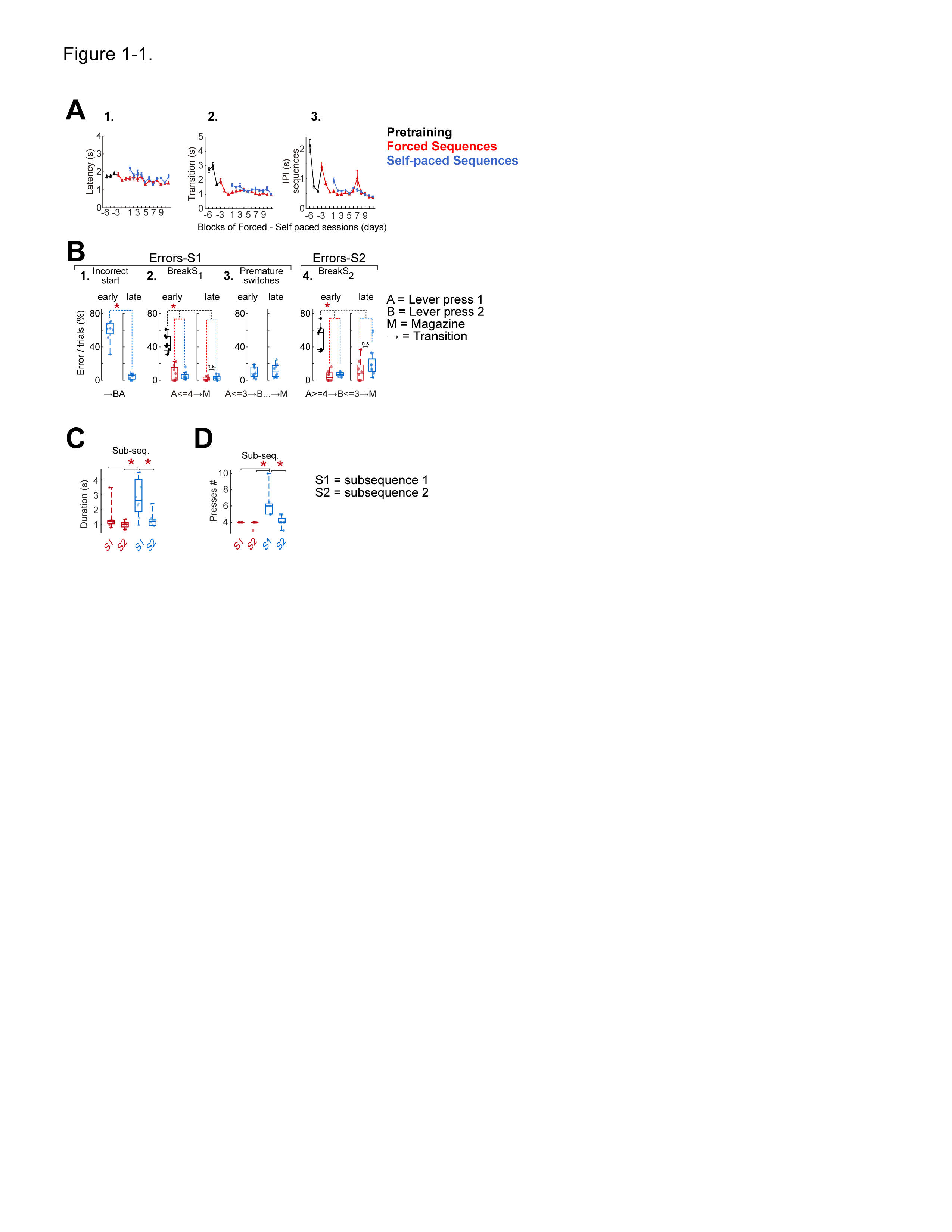

Supplement: Extended Data Figure 1-1 — Extended data from Figure 1. A, Latency to start the sequences (1), transition time between the last press in the subsequence 1 to the first press in the subsequence 2 (2), and the press intervals intrasequences (3) along days. B, The different categories of errors that mice executed while performing forced or self-paced sequences early and late in training. C, D, Mean of duration and lever presses per individual subsequences. Data from WT animals (n = 8); *p < 0.05, Mann–Whitney U test. Download Figure 1-1, TIF file. [file enu-eN-NWR-0173-21-s01.tif]

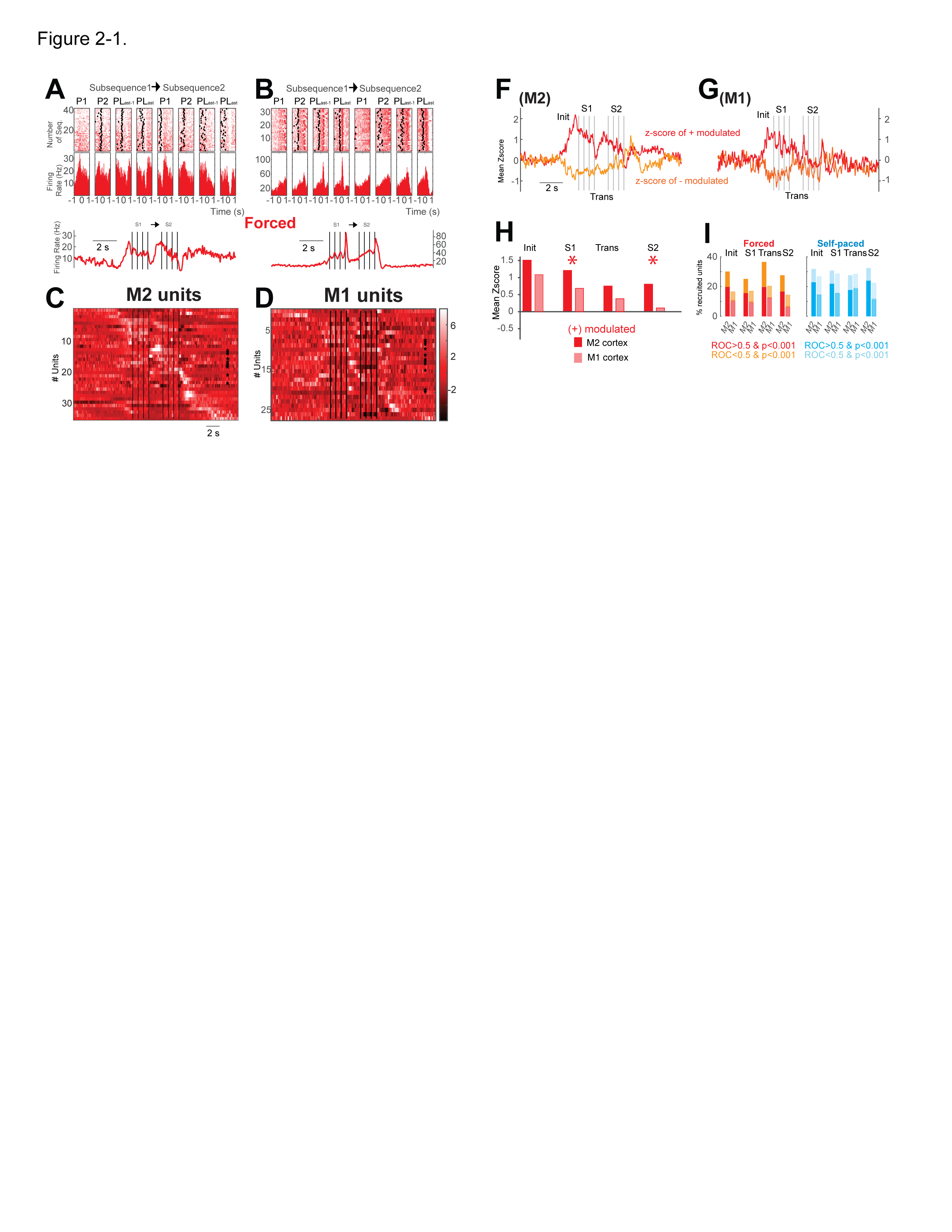

Supplement: Extended Data Figure 2-1 — Activity modulation in premotor and motor cortical neurons during the execution of forced sequences of actions. A, B, Raster plots and perievent histogram from an M2 or an M1 unit, respectively, aligned to the first (P1), second (P2), penultimate (PLast-1), and last lever press (PLast) of S1 and S2 for forced sequences. Bottom panels, Mean firing rate from the upper panels. C, D, Z score of individual units. F, G, Mean z score from the units recorded that presented significant modulation 1 s before the initiation of the sequence from M2 or M1. H, Comparison of the mean z score from F, G, M2 (dark red) versus M1 (light red) for positively modulated units. I, Percentage of recruited units (bins of 200 ms, a sliding window of 10 ms) for M2 versus M1 during forced and self-paced sequences; *p < 0.05, z score test. Init. = initiation, S1 = subsequence 1, Trans = transition, S2 = subsequence 2. Download Figure 2-1, TIF file. [file enu-eN-NWR-0173-21-s02.tif]

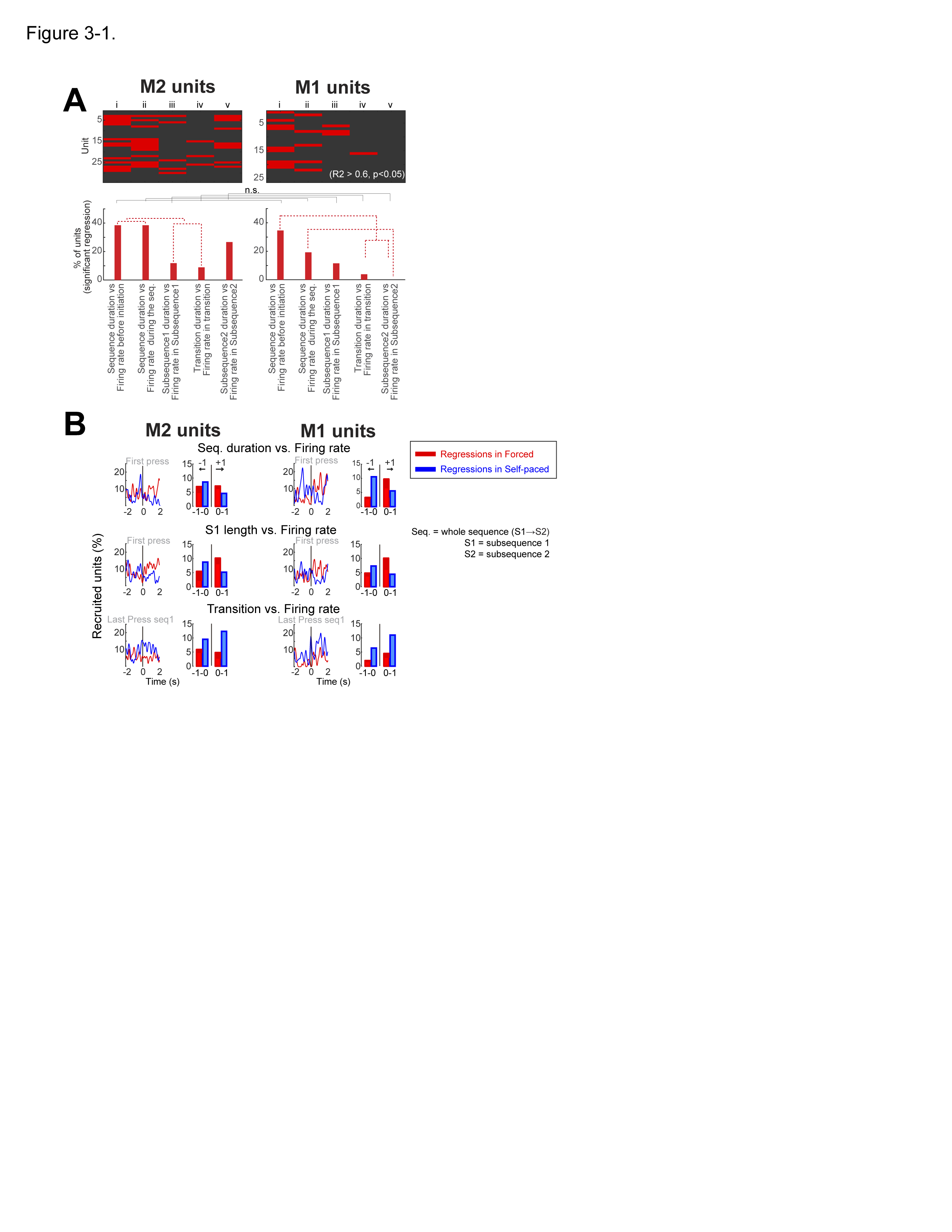

Supplement: Extended Data Figure 3-1 — Both M2 and M1 contain units encoding the temporality of the forced sequences of actions. A, top row panels, Significant regression analysis per unit columns i, between the firing rate (FR) 1 s before the start of the sequences (Seq) versus the duration of the sequences, columns ii: FR during the sequences versus duration of sequences, columns iii: FR in the subsequence 1 (S1) versus the duration of S1, columns vi: FR during the transition versus the transition time, and columns v: FR during the subsequence 2 (S2) versus the S2 duration. Bottom panels, The proportion of units that presented significant regression (R2 > 0.6 and p < 0.05). The dashed lines depict comparisons with χ2 test. Corrections for multiple comparison was considered (see Materials and Methods). B, Percentage of recruited units (presenting significant regression, bins of 200-ms sliding window of 10 ms regarding the tittles in each plot). Bars show the average of the recruited units 1 s before and after the start of the sequences except for the transitions, which is aligned to the last press of the subsequence 1. Download Figure 3-1, TIF file. [file enu-eN-NWR-0173-21-s03.tif]

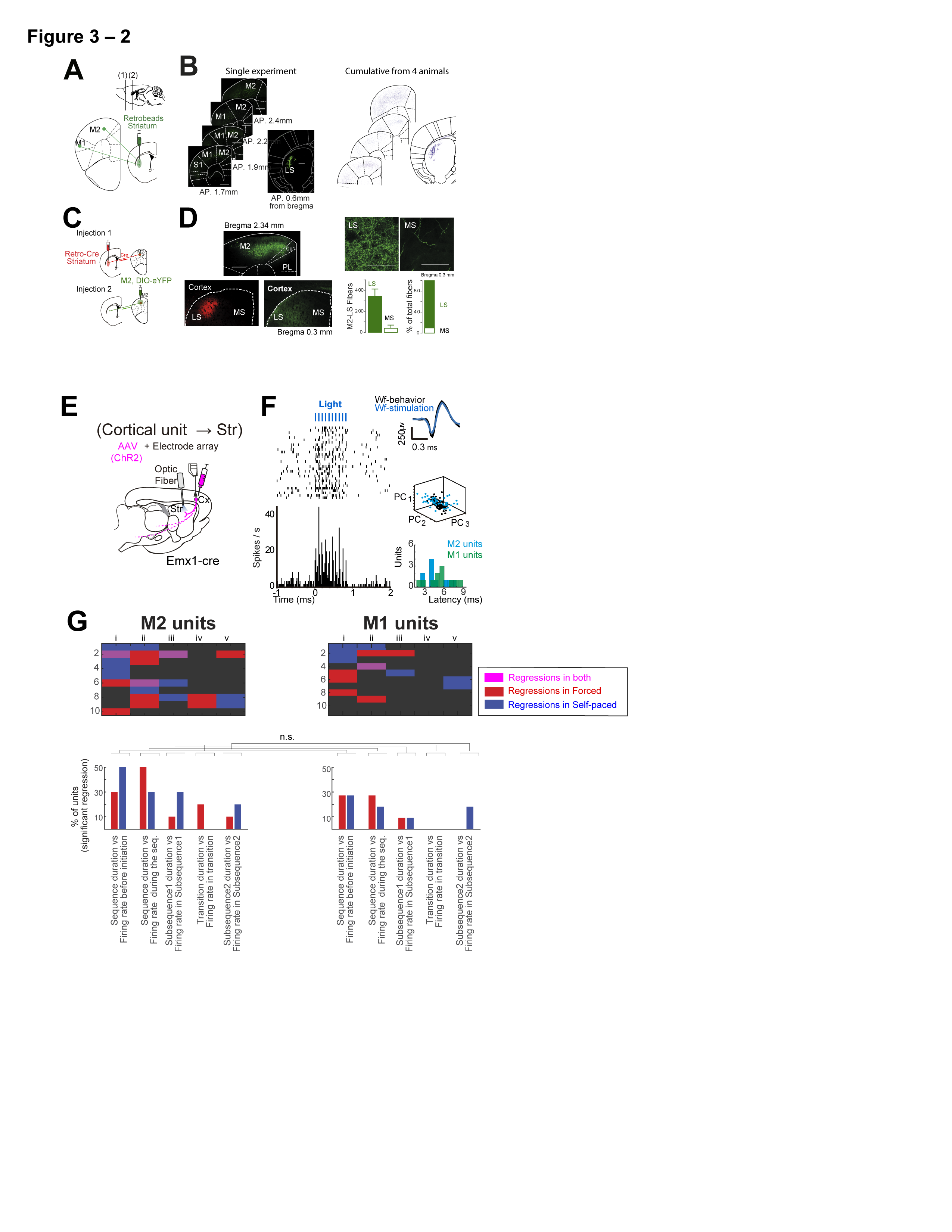

Supplement: Extended Data Figure 3-2 — Confirmation of the M2-M1 cells and their projections into the LS and linear regressions between the activity and the temporal parameters of the execution of sequences from the photo-identified M2 or M1 cortico-striatal neurons. A, Scheme of retrograde tracing of M2 and M1 neurons (left) labeled by retrobead injection into the LS (right). B, left panels, Anteroposterior coronal photomicrographs showing retrobead labeled cells in M2/M1 (left) from the retrobead injections into the LS. Scale bar: 500 μm. Right panels, Diagrams presenting summed data from four animals that were injected with retrobeads into the LS in binary color code. C, Scheme of premotor cortex anterograde tracing using AAV-Retro-Cre-mCherry injection into the LS (injection1) and AAV-DIO-eYFP into the premotor cortex (M2; injection2). D, Left photomicrographs depict an example from one animal of the experiment described in C. Prelimbic cortex (PL), cingulate cortex (Cg1). Scale bar: 500 μm. Right top panels, Zoom-in of the LS and the medial striatum (MS) showing the premotor cortico-striatal projections labeled in green. Scale bar: 50 μm. The bars show the quantification of the M2→LS fibers reaching the LS at AP. 0.3 from bregma (coordinate where most of the fiber tips of the optogenetic inhibition were found). E, Diagram of the injection site to express ChR2 into M1 or M2 cortex and their projections, the electrode array (M2 or M1 cortex), and the optical fiber implantation (LS). F, Raster-plot example of a cortico-striatal PID unit and the perievent time histogram aligned at the start of the first pulse of a train of blue light (10 Hz; 2 mW, 473 nm). Right panels, Mean waveforms, principal component analysis, and latency to response to stimulation during behavior (black) and photo-identification protocol (blue). G, top row panels, Significant regression analysis per unit; columns i, between the firing rate (FR) 1 s before the start of the sequences versus the sequences duration; columns ii, FR dur [file enu-eN-NWR-0173-21-s04.tif]

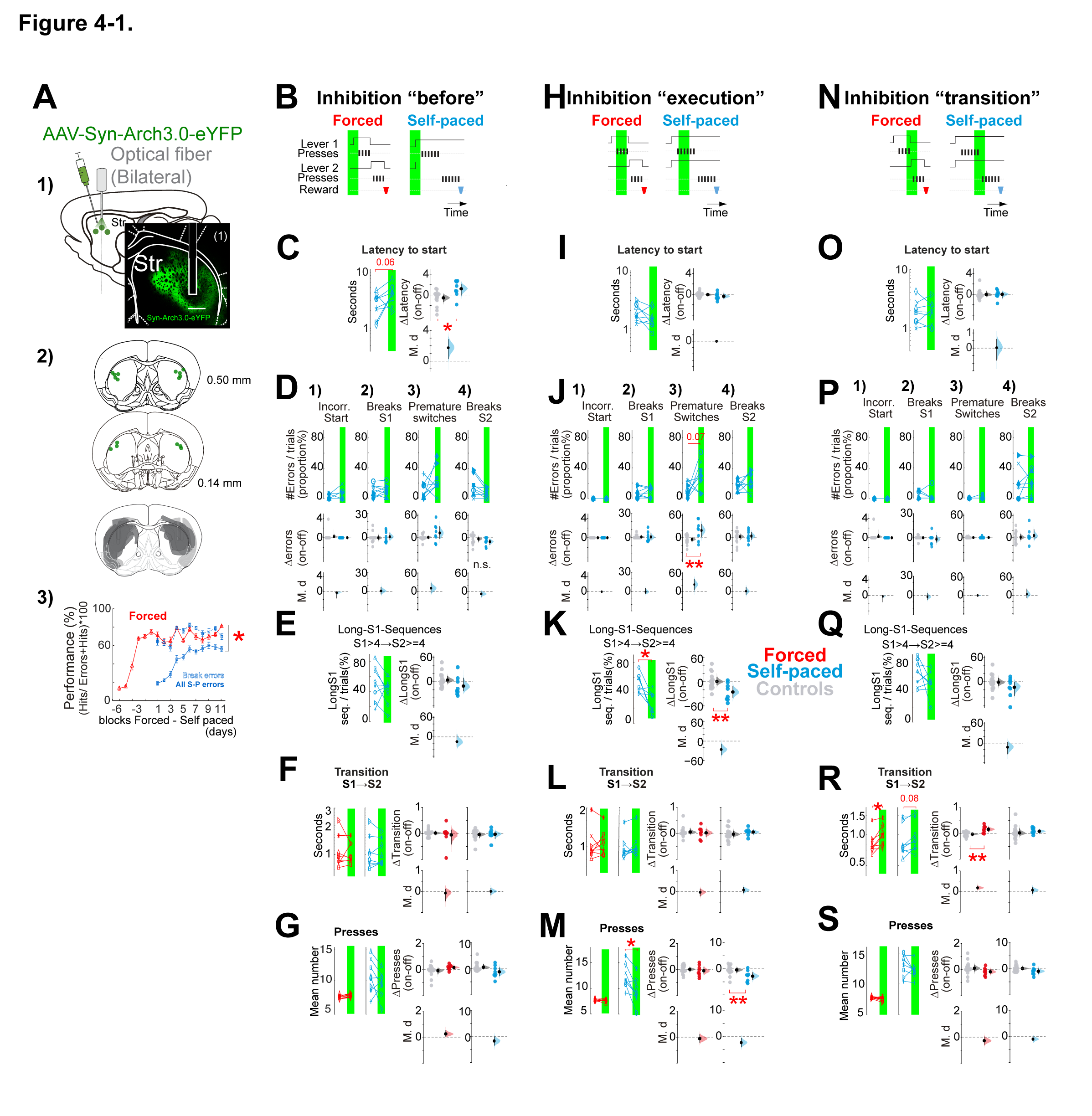

Supplement: Extended Data Figure 4-1 — Inhibition of the lateral striatal neurons impairs the initiation and execution of sequences of actions. A, panel 1, Diagram of the injection of archaerhodopsin 3.0-eYFP (under the synapsin promoter; Syn-Arch3.0) into the LS and the corresponding site for fiber optic implantation. The photomicrograph shows a coronal section at the striatal level depicting the Arch3.0-eYFP expression of a single animal. Panel 2, Location of the fiber optic tips into the LS from the eight animals considered for this group (green points). The coronal section with the shadows in grey represents the extension of the Arch3.0 expression. Panel 3, Percentage of correct sequences [correct/(errors + correct)] along with the training. B, H, N, Schemes of the inhibition protocols applied in the corresponding columns. Green shadows depict 2 s of continuous light, forced (red), and self-paced sequences (blue). B, Inhibition before the initiation, triggered by the breaking of an infrared bean placed outside of the magazine towards the lever press. H, Inhibition triggered by the first press in the execution. N, Inhibition triggered by the penultimate press in the subsequence 1 of the sequences. C, I, O, Effect of inhibition of lateral striatal neurons on the latency to initiate forced or self-paced sequences. D, J, P, E, upper panels 1–4, Quantification per animal in off versus on trials of the proportion of each category of error. E, K, Q, As in C, I, O evaluating the proportion of Long-S1 sequences (S1 > 4→S2 >= 4). F, L, R, As in C, I, O evaluating the transition time in forced and self-paced sequences. G, M, S, As in C, I, O evaluating the number of presses in forced and self-paced sequences. In each paired graph, each line plots the mean effect per animal during trials of optogenetic inhibition (on; green shadow) versus trials without inhibition (off; no shadow) from the same session. In panels C–S, Δ on-off panels are obtained from the mean difference per animal in the on-off trials, adding t [file enu-eN-NWR-0173-21-s05.tif]

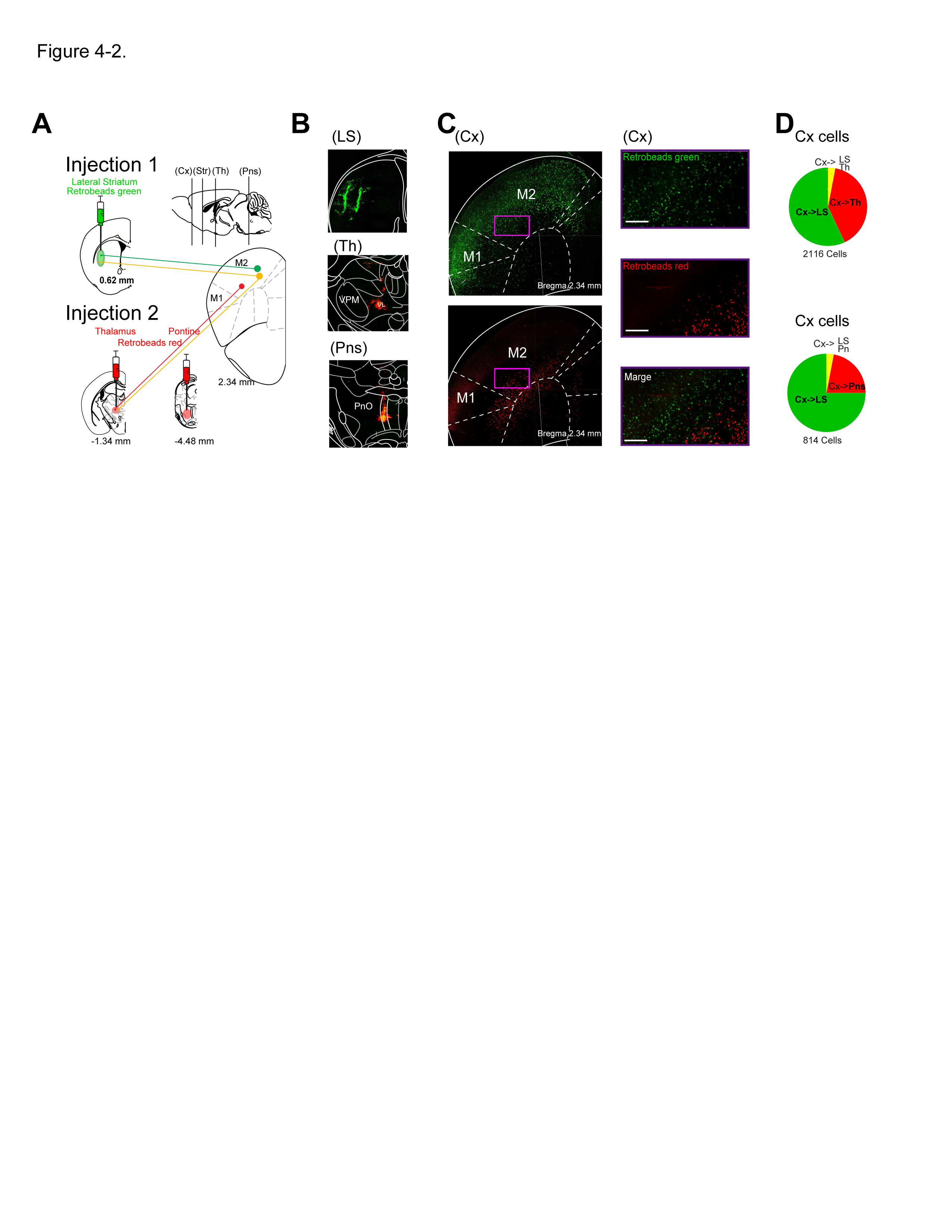

Supplement: Extended Data Figure 4-2 — M2/M1 retrogradely labeled cells from the LS, the thalamus (Th), and the pons (Pns). A, Diagram of the experiment to trace corticofugal projections to the thalamus or to the pons leaving collaterals/crossing by the LS. Top left, First retrograde tracer injection (green retrobeads) into the LS. Bottom panels, Second retrograde tracer injection (red retrobeads) into the thalamus or the pons. Top right, Diagram of a sagittal section showing the anteroposterior level of the striatum, the thalamus or the pons (black vertical lines). Bottom right, A coronal diagram illustrating the M2 cortical projection to the LS (green), the thalamus (red), or both (yellow). B, Photomicrographs of the sites of injection of retrobeads into the LS (green) or the thalamus (Th; red) or the pons (Pns; red). C, left panels, green, M2/M1 cortico-striatal cells; red, M2/M1 cortico-thalamic cells. Right panels, Zoom-in from the pink squares on the left. D, Percentage of single target cells and cells reaching either the thalamus or the pons leaving collaterals/crossing at the LS. Green, The proportion of cells only targeting the LS; red, the proportion of cells targeting either the thalamus (top) or the pons (bottom); yellow, the proportion of cells that targeted the LS and the thalamus (top) or the LS and the pons (bottom) simultaneously. Download Figure 4-2, TIF file. [file enu-eN-NWR-0173-21-s06.tif]

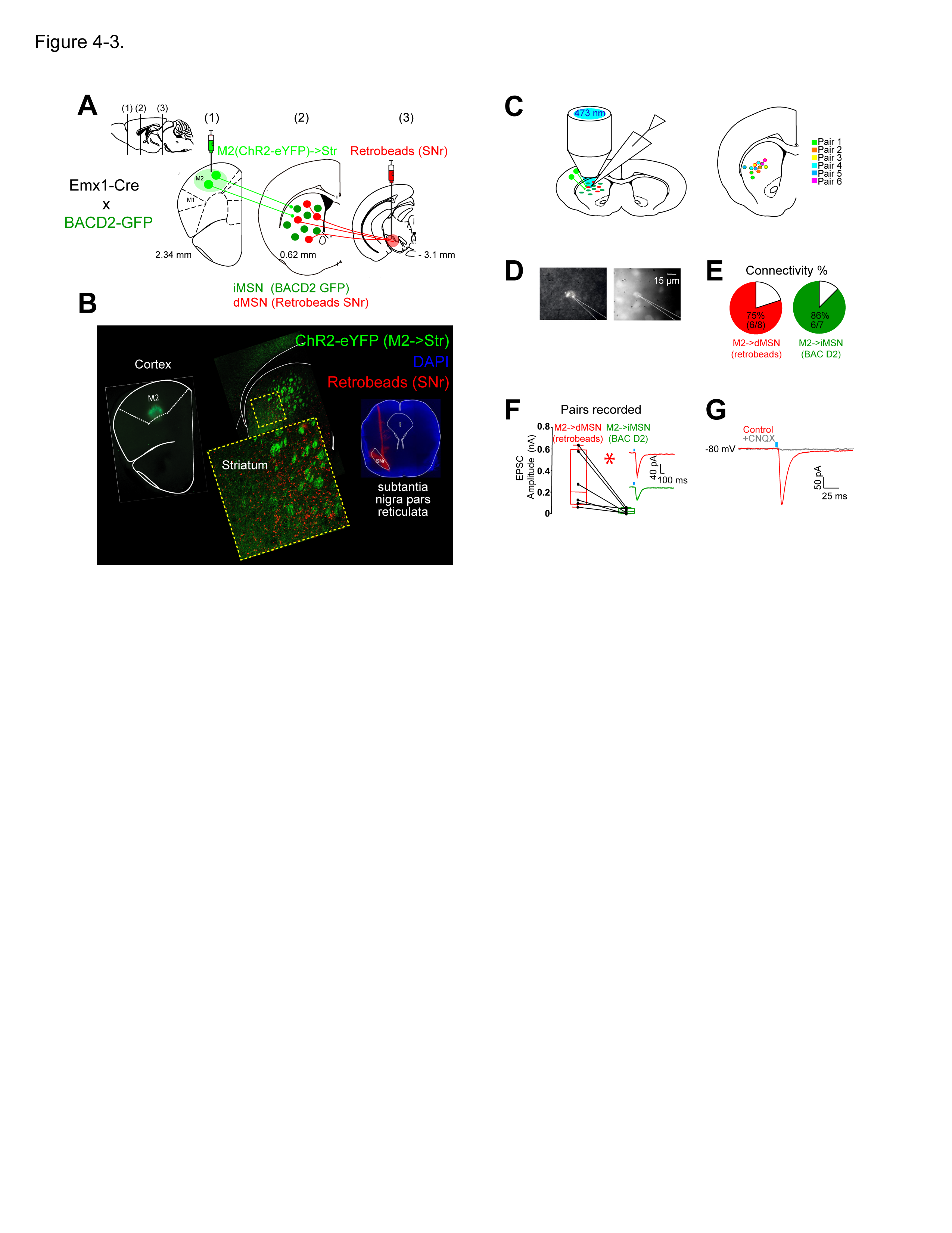

Supplement: Extended Data Figure 4-3 — Premotor cortico-striatal projections innervate the direct pathway with a stronger synaptic weight than the indirect pathway. A, Diagram of ex vivo experiments to record the cortico-striatal synaptic connectivity on the two kinds of striatal projection neurons, striatopallidal (iMSN) or striatonigral (dMSN). (1) Coronal diagram showing the injection of ChR2 into premotor cortex (M2). (2) Coronal diagram showing the premotor cortex projections onto iMSN (green dots) or dMSN (red dots). (3) Coronal diagram showing the injection of retrobeads into the SNr. B, left, Photomicrograph of ChR2 injection into M2. Middle, A photomicrograph of the lateral part of the striatum showing the premotor cortex projections (eYFP; green) and the dMSN labeled with retrobeads (red). Right, Photomicrograph of retrobeads (red) injection into SNr. C, left, Diagram of stimulation with blue light on the LS to produce EPSCs on the MSN recorded by whole-cell patch clamp. Right, Location of pairs of cells recorded in the same slice into the striatum (from four animals). Each color depicts one pair of recordings. D, Photomicrographs of a dMSN (left) and an iMSN (right). White lines depict the recording electrode. E, The proportion of dMSN or iMSN cells that presented EPSCs in response to blue light stimulation of the M2→striatal-ChR2 axons. F, Mean amplitude of EPSCs (pA) recorded on a dMSN (red) and iMSN (green). Inset, Representative EPSCs recorded in response to optogenetic stimulation of M2→striatal-ChR2 axons. The holding potential was –80 mV; the shaded region indicates SEM; *p = 0.02, Wilcoxon test. G, Example of EPSCs recorded on a dMSN blocked by the AMPA antagonist CNQX. Download Figure 4-3, TIF file. [file enu-eN-NWR-0173-21-s07.tif]
